# A complete guide to human microbiomes: Body niches, transmission, development, dysbiosis, and restoration

**DOI:** 10.3389/fsysb.2022.951403

**Published:** 2022-07-22

**Authors:** Jelissa Reynoso-García, Angel E. Miranda-Santiago, Natalie M. Meléndez-Vázquez, Kimil Acosta-Pagán, Mitchell Sánchez-Rosado, Jennifer Díaz-Rivera, Angélica M. Rosado-Quiñones, Luis Acevedo-Márquez, Lorna Cruz-Roldán, Eduardo L. Tosado-Rodríguez, María Del Mar Figueroa-Gispert, Filipa Godoy-Vitorino

**Affiliations:** 1 Department of Biology, UPR Rio Piedras Campus, San Juan, PR, United States; 2 Department of Microbiology and Medical Zoology, UPR School of Medicine, San Juan, PR, United States

**Keywords:** human microbiome, body niches, evolution, dysbiosis, restoration

## Abstract

Humans are supra-organisms co-evolved with microbial communities (Prokaryotic and Eukaryotic), named the microbiome. These microbiomes supply essential ecosystem services that play critical roles in human health. A loss of indigenous microbes through modern lifestyles leads to microbial extinctions, associated with many diseases and epidemics. This narrative review conforms a complete guide to the human holobiont—comprising the host and all its symbiont populations- summarizes the latest and most significant research findings in human microbiome. It pretends to be a comprehensive resource in the field, describing all human body niches and their dominant microbial taxa while discussing common perturbations on microbial homeostasis, impacts of urbanization and restoration and humanitarian efforts to preserve good microbes from extinction.

## Introduction

Metagenomics and its applications have revolutionized microbiology, medicine, and our contemporary lifestyles. The capacity to sequence microbes from all sample types and the multiple advantageous public computational pipelines and tools, have made microbiome studies accessible to most scientific fields. The study of communities of microbiological organisms directly in their natural settings is a branch of genomics that sprang out of the Human Genome Project, and is continuously revealing fascinating frontiers of knowledge to better understand health and disease (Turnbaugh et al., 2007).

All body niches are colonized by a microbiome which is composed by the components of the tree of life from all domains, Eukarya, Bacteria, Archaea, and viruses. They all make up the human body, and this collective domain results in different phenotypes. Animals are not simply individuals by the physiology criterion, but given the variety of symbionts in direct contact with the hosts, there are additional and unique metabolic pathways providing other important physiological functions.

For many, the host and its associated microbiome is considered a human organ (Baquero and Nombela, 2012) or a biological individual altogether (Gilbert et al., 2012), to others it constitutes an ecosystem (Foster et al., 2017), or even a unit of selection, upgrading and expanding fundamentally unshaken theories such as Darwin’s evolution principles—with the inclusion of the Hologenome Concept (Zilber-Rosenberg and Rosenberg, 2008). This term, still raises discussion on the individuality of the holobiont (multicellular host and its associated microbiome) (Bordenstein and Theis, 2015; Suárez and Stencel, 2020). Eukaryotes, in their complexity, are not independent individuals, but rather natural units with associated symbionts and their metagenomes.

The microbiota, like other organs, is inherited through the dynamics of birth, with different dependent outcomes. It evolves with the host throughout his life, and we now know that lifestyle choices have great impact to its homeostasis. This new revelation has changed how we view Biology and has greatly broadened our knowledge of biodiversity and the multi-kingdom interactions responsible for health and disease. Thus, the goal of this narrative review is to summarize what is known of the human microbiome, including recent and detailed literature on all body niches, with a special emphasis in studies of the gastrointestinal tract, microbial transmission, and ongoing restoration efforts that could provide relief to many diseases.

## Evolution, transmission, and development of human microbiomes

**FIGURE 1:**
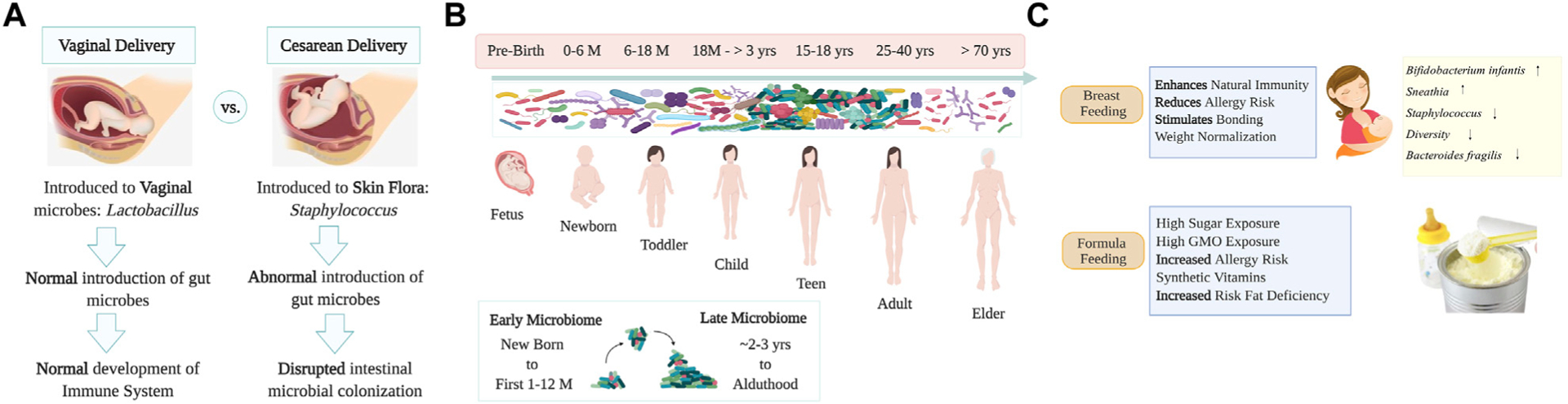
Human microbiome development and transmission. Panel **(A)** Representation of humans’ first contact with microbes during birth and a comparison of how delivery mode (vaginal delivery and C-section) impacts infant microbiota. Panel **(B)** Human microbial transmission and development from pre-birth to adulthood. Microbes that colonize newborns will form a variety of niches in different body sites, adult diversity is attained at ~3 years old. Elder people have a decrease in microbial diversity leading to dysbiosis that may be associated with neurodegenerative disorders. Panel **(C)** Practices of breastfeeding and/or formula feeding play an important role in shaping the intestinal microbiome. Formula feeding is associated with intestinal inflammation, with an increase in *Enterobacteriaceae* and *Clostridium* spp. and reduced levels of probiotic *Bifidobacterium* and *Lactobacillus* spp. acquired *via* lactation. Image created with BioRender.com.

Humans depend on their microbes for health. As we live in a microbial world, human life must be framed in the context of microbial evolution (McFall-Ngai et al., 2013). The microbiome indeed performs a critical role in maintaining human health (Godoy-Vitorino, 2019). The holobiont concept -a term initially coined by Margulis—theorized an interaction between host cells and their associated microbial communities, and such unit undergoes natural selection, which drives the features of these host-symbiont associations (Margulis, 1990; Margulis and Fester, 1991) and even preceding work from the 19th century by German botanist Karl Brandt, already theorized that self-formed chlorophyll was supposedly absent in animals and likely due to “invading plants” which kept “physiological independence” (Brandt, 1881), as described and discussed recently by others (Suárez, 2018; Baedke et al., 2020). The Developmental Origin of Health and Disease (DOHaD) theory is based on the concept that the origins of the lifestyle-related disease occur pre-birth, at the embryonic, fetal, and neonatal stages due to the interrelation between genes environment and lifestyle (nutrition, stress, or chemical cues) (Mandy and Nyirenda, 2018). Indeed, the first contact humans have with microbes is at birth, and since these early beginnings, microbes sustain life and development (Funkhouser and Bordenstein, 2013; Chiu and Gilbert, 2015; Dominguez-Bello et al., 2019). Bacterial transfer from mother to infant occurs when babies go through the vaginal canal, or via skin contact by C-section at birth, and by skin-to-skin contact during breastfeeding (Dominguez-Bello et al., 2010). Only a group of the microbes to which the newborn is initially exposed at birth will permanently colonize various body niches (Figure 1). Vaginal Lactobacilli have long been the keystone species of genital communities in reproductive-age women and are passed down to newborns born vaginally, contributing to milk digestion. In turn, babies who are delivered by Cesarean section (C-section) are often colonized by bacteria that are more commonly found on the skin, including *Staphylococcus*, *Propionibacterium* or *Corynebacterium*, often coming from the hands of medical workers (Dominguez-Bello and Godoy-Vitorino, 2013; Dominguez-Bello et al., 2019). Thus, skin bacteria also play a crucial role during vertical microbial transmission in the development and maturation of the future microbiome of babies born *via* C-section. The mode of transmission is likely to be part of a response to protect and promote fetus health before exposure to other environmental conditions and microbes. Practices such as C-section, perinatal antibiotics, and formula feeding have been linked to increased risks of metabolic and immune diseases related with dysbiosis (Mueller et al., 2015). The medicalization of birth in many developed countries has transformed the quality of contact between mothers and newborns, altering this initial microbiome transmission (Mueller et al., 2015). More than 30% of all live births in the United States (US) were performed by C-section, 26.9% in Europe, ~44.3% in Latin America (55% in Brazil and 58% in the Dominican Republic, one of the highest rates in the world) and 21.1% globally (Zakerihamidi et al., 2015). C-section was first introduced to reduce the risks for the mother and the fetus. Society has accepted that this medical procedure is painless, safer, and sometimes healthier than vaginal delivery (Delport, 2019). Increasing evidence suggests that avoiding exposure to the maternal flora during natural labor or vaginal birth, adversely affects gut function and immune system development, increasing the risks of obesity, asthma, allergies, and autoimmune diseases (Black et al., 2016). There are also changes in the microbiome of newborns when delivered in a hospital or home environment. Those born in the hospital resemble some of the reported effects of other stressors such as C-section, antibiotics, or formula feeding, with a reduction of *Bacteroides*, *Bifidobacterium*, and *Ruminococcus* and an increase in *Enterobacteria* and *Clostridium* species (Black et al., 2016). Hospitalizations related to perinatal interventions and mode of delivery also affect microbial transmission—causing effects that persist in the intestinal microbiota of infants 1 month after birth (Combellick et al., 2018). Medical environments are spaces both designed and managed to minimize negative impacts on patient health. However, studies have reported bacterial presence in operating rooms (OR) in which fecal-like bacteria accumulate mostly on the floor but also on the walls might negatively impact the newborn (Derilus et al., 2020). Breast milk contains important developmental and immune-promoting factors such as oligosaccharides, immunoglobulins (IgA), and lactoferrin which protect the newborn passively and actively against excessive intestinal inflammation (Gregory et al., 2016). Bacteria acquired during lactation include lactic acid producers, who commonly digest lactose, and other organisms that utilize the milk glycans known as Human Milk Oligosaccharides (HMOs). These HMOs, which are indigestible for neonates, can shape the infant’s gut microbial composition, selecting for *Bifidobacterium* spp. and *Lactobacillus* spp. (Dominguez-Bello et al., 2019). Such changes to the microbiota provide colonization resistance against common opportunistic pathogens like Enterobacteria and *Clostridia* (Figure 1). On the contrary, children fed formula have an increased risk for obesity, higher diversity, and enrichment of Bacteroidaceae in 1 year of age. Although the microbiota in neonates is established at birth, it will shape throughout the next 3 years of life due to environmental factors such as diet, antibiotics, hygiene, and the built environment (Indiani et al., 2018). Antibiotics are known to decrease the overall diversity of the infant’s microbiota and aid in the selection of drug-resistant organisms (Reyman et al., 2022). Infants treated with antibiotics tend to have lower bacterial diversity as well as an increase of Enterobacteriaceae and *Enterococcus* (Reyman et al., 2022). The early use of this treatment has been associated with higher risks of allergic diseases (Zwittink et al., 2018), eczema (Kim et al., 2019a), and obesity (Schulfer and Blaser, 2015), and type 1 diabetes (Langdon et al., 2016). Another factor that heavily contributes to the neonatal overall microbial content is household animals (Kim et al., 2019a). Exposure to pets increases the abundance of *Ruminococcus* and *Oscillospira* species, which may protect against allergic disorders and obesity in children (Tun et al., 2017). Exposure to other modern lifestyle factors, including in-utero exposure to stresses such as hurricanes, or other extreme weather events, have been explored and shown to impact the microbiota of infants (REF: https://www.sciencedirect.com/science/article/pii/S2772829322000352).

## Human body niches: A glance from the simple to more complex

### The vaginal microbiome

The vagina is the microbial organ with the least diversity in the human body, with a dominance of *Lactobacillus*, a species that impedes the colonization of other bacteria that would otherwise cause infections. The lactic acid produced by the Lactobacilli provides a protective role by maintaining an acidic pH (<4.5); serving as a chemical barrier (Valenti et al., 2018). In addition, *Lactobacillus* spp. produce bacteriocins, H_2_O_2,_ and reactive oxygen species (ROS), impeding the colonization and adherence of pathogens (Felten et al., 1999), organisms that would otherwise cause recurrent vulvovaginal infections (RVVI) associated with discomfort, odor, discharge, infertility, and, if pregnant, could even lead to miscarriages. The vaginal microbiota can be characterized in five Community State-Types (CST), representing different microbial groups (Zhou et al., 2007). CST-I has a predominant abundance of *L. crispatus,* CST-II has *L. gasseri*, CST-III has *L. iners*, and CST-V has *L. jensenii*. CST-IV, on the other hand, has a reduction of *Lactobacillus* spp. and a higher abundance of anaerobic bacteria such as *Prevotella*, *Atopobium*, *Sneathia*, and *Gardnerella*, which have been associated with bacterial vaginosis (Di Paola et al., 2017). CST profiles in women are known to vary by ethnicity. Caucasians tend to exhibit a CST-I dominated microbiota, while African-American and Hispanic women tend to present a CST-IV profile (Di Paola et al., 2017). Having a non-*L*. *crispatus* dominant community does not necessarily mean severe dysbiosis; studies have shown healthy Latinas who have a *L. iners* dominant community can be asymptomatic (Godoy-Vitorino et al., 2018; Vargas-Robles et al., 2020). Hormonal changes across the reproductive cycle in women can disrupt the microbial equilibrium. When there are high hormonal levels, the abundance of glycogen increases in the vagina, which is used by bacteria to promote an increase in diversity (Kaur et al., 2020). At the same time, glycogen is used by *Lactobacillus* spp. to produce lactic acid, reducing and stabilizing the diversity present. Puberty, menstruation, pregnancy, and menopause compose the main stages of the female whole reproductive cycle. The cervicovaginal microbiota becomes even more dominated by *Lactobacillus* during pregnancy, resulting in less diversified profiles than in non-pregnant women (Serrano et al., 2019). However, as estrogen and progesterone levels fall after menopause, there is a drop in *Lactobacillus* spp. and an increase in vaginal pH (Auriemma et al., 2021).

### The skin microbiota

**FIGURE 2:**
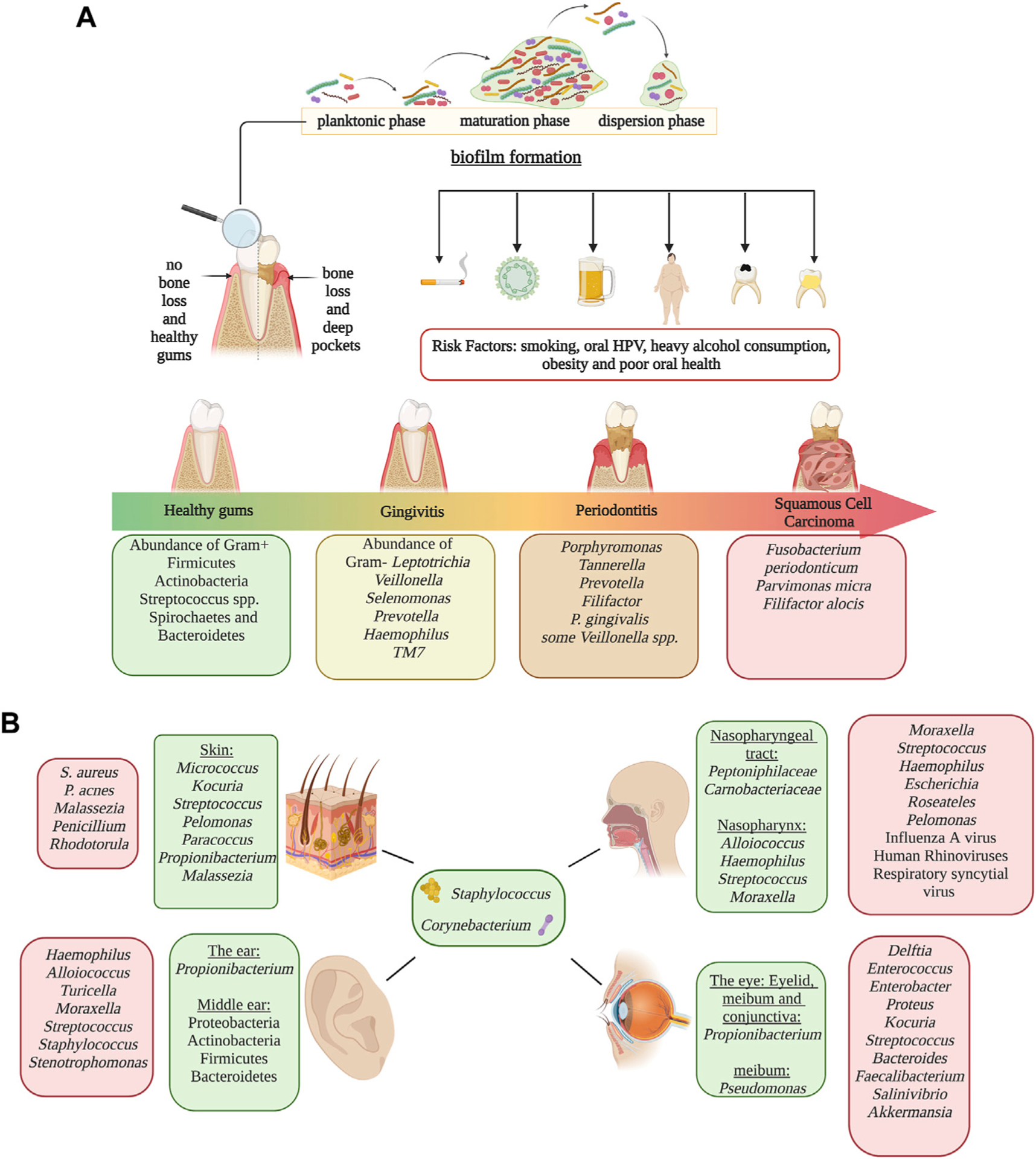
Microbiome in human epithelial and mucosal sites other than the gut. Panel **(A)** Representation of biofilm formation in dental plaque, periodontal disease progression and risk factors associated with periodontitis and oral cancer at each progression stage. Disease development is associated oral dysbiosis. Disease stages are identified by colors; green represents healthy gums, yellow represents gingivitis, orange represents periodontitis and red represents oral squamous cell carcinoma. Panel **(B)** Characteristic microbiome of the skin, ear, eyes and the nasopharyngeal tract. Homeostatic microbiome are identified in green, and microorganisms that increase in abundance during dysbiosis are identified in red. Alterations of microbial populations can lead to the development of different health conditions which could be irreversible. Image created with BioRender.com.

The skin is an essential element of defense against pathogens. Its physiological and anatomical properties change throughout the body, shaping microbial composition. Compared to the most diverse body sites, the skin microbiome has fewer taxa due to its textural characteristics such as oil, moisture, sebaceous glands, and acidic pH (Chaudhari et al., 2020). External factors implicated in changes in the skin microbiota include the use of antibiotics (Chien et al., 2019), cutaneous burns (Sanjar et al., 2020), skincare, and hygiene products (Bouslimani et al., 2019), and lifestyle habits (Blaser et al., 2013). The most dominant genus in the skin is *Staphylococcus*, *Propionibacterium*, *Corynebacterium*, and *Streptococcus* (Bay et al., 2020). Furthermore, oilier sites have significant dominance of *Propionibacterium* species (lipophilic), whereas in humid niches, *Staphylococcus* and *Corynebacterium* species thrive (Bay et al., 2020). Fungi are also a major component of the microbiome. For example, *Malassezia* is a major lipophilic yeast distributed throughout the body; however, other fungi are site-specific such as *Aspergillus* spp., *Cryptococcus* spp., and *Rhodotorula* spp. who colonizes regions of the feet (Gupta and Kohli, 2004; Jo et al., 2017). In addition, shifts in fungal composition can occur with age, as children have a marked profile of Ascomycetes and lower levels of *Malassezia* when compared to adults. The age-associated differences in the skin microbiome is so marked that it has been used to predict an individual’s age with a range of approximately 4 years. Dysbiosis of the skin microbiome has been related to skin diseases/conditions. For instance, patients with psoriasis have a higher abundance of Proteobacteria and *S. aureus* and a decrease in *Acinetobacter* when compared to healthy individuals (Chang et al., 2018). Similarly, the skin of patients with atopic dermatitis is characterized by a higher prevalence of *S. aureus* (Paller et al., 2019). *S. aureus* and *S. epidermidis* distinguished skin lesions of patients with systemic lupus erythematosus (SLE), whereas healthy individuals had a higher abundance of *Cutibacterium* (Huang et al., 2020). On the other hand, patients with alopecia have an increase in *P. acnes* and a decrease in common members of the skin microbiome such as *Propionibacterium*, *Corynebacterium*, and *Staphylococcus* (Ho et al., 2019) (Figure 2).

### The eye microbiota

The eye has three major microbial niches: the eyelid skin, meibum, and conjunctiva, which differ in diversity and composition. Similar to the skin microbiome, the microbial composition of the eyelids is dominated by two skin taxa *Staphylococcus* and *Propionibacterium* (Suzuki et al., 2020). The meibum is characterized by a dominance of *Propionibacterium* and *Pseudomonas,* while the conjunctiva is defined exclusively by *Propionibacterium* (Suzuki et al., 2020). The dysbiosis in the conjunctiva microbiome has been associated with different health conditions, such as keratoconjunctivitis, mucosa-associated lymphoid tissue (MALT) lymphoma, and high glucose levels on the ocular surface due to diabetes (Asao et al., 2019). Notably, the microbiota of conjunctiva in MALT lymphoma patients is dominated by *Delftia* (Asao et al., 2019). When evaluating the role of the ocular microbiota in relation to diabetes, mice studies reveal a reduced diversity in Type 2 diabetes (Li et al., 2019; Suzuki et al., 2020), while an increase in *Bacteroides* and a decrease in Proteobacteria and Acinetobacter are observed in Type 2 diabetes (T2D) (Li et al., 2019).

### The ear microbiota

The microbiota of the ear canal is similar to that found on the skin. Therefore, *Corynebacterium*, *Staphylococcus*, and *Propionibacterium* genera are prevalent taxa (Jervis-Bardy et al., 2019). There is still a debate over whether microbes from the nasopharynx colonize the middle ear or if this is a sterile site. Although previous findings suggested no microbial colonization, a recent Illumina microbiome profiling study demonstrated that the middle ear is actually colonized by Proteobacteria, Actinobacteria, and Firmicutes (Jervis-Bardy et al., 2019). Otitis media infections can be characterized as Acute Otitis Media (AOM) or Chronic Otitis Media with Effusion (COME). Dysbiotic changes are observed in adults and children who suffer from Otitis Media inflammation (Lappan et al., 2018). The pathogenesis and development of AOM are dependent on the microbiome of the nasopharynx, with *Haemophilus*, *Alloiococcus*, *Staphylococcus*, *Turicella, Moraxella*, and *Streptococcus* being taxa normally associated with this condition (Lappan et al., 2018). In COME patients, it’s been documented that a higher abundance of *Alloiococcus*, *Haemophilus*, *Moraxella*, *Turicella*, *Stenotrophomonas*, *Streptococcus*, and *Staphylococcus* (Kolbe et al., 2019). It’s important to mention that *Alloiococcus* and *Turicella* are not found in the healthy middle ear. In addition, COME is associated with respiratory illnesses such as asthma and bronchiolitis while reflecting a lower richness and evenness in comparison with those patients that do not present lower respiratory diseases (Kolbe et al., 2019).

### The microbiota of the nasopharyngeal tract

The nasopharynx is a component of the upper respiratory tract, specifically located at the upper part of the throat behind the nose. The microbiome of the nasal cavity mucosa is colonized primarily by *Corynebacteriaceae* and *Staphylococcaceae* families, while *Peptoniphilaceae* and *Carnobacteriacea* are in lower abundance (Kang and Kang, 2021). This niche also has a high abundance of *Staphylococcus*, *Corynebacterium*, *Alloiococcus*, *Haemophilus*, *Streptococcus*, *Granulicatella*, and *Moraxella* (Teo et al., 2015). Diseases such as asthma, influenza A virus (IAV), bronchiolitis, and rhinosinusitis acute respiratory illness (ARI) are all associated with changes in the microbiota. Children with IAV infection have increased microbial diversity, specifically of *Streptococcus*—associated with the production of type I interferons during IAV infection, with a concomitant decrease in *Corynebacterium, Moraxella* and *Dolosigranulum* (Wen et al., 2018). Infants with bronchiolitis have an increasing dominance of *Haemophilus*, *Moraxella*, and *Streptococcus* when compared with healthy individuals (Stewart et al., 2017). Chronic rhinosinusitis (CRS) is also a dysbiosis-related disease, with higher alpha diversity in CRS compared to healthy individuals and increasing levels of Proteobacteria and *Escherichia*. Other genera, including *Roseateles*, *Pseudomonas*, and *Escherichia*, were positively correlated with CRS symptom severity (Copeland et al., 2018).

### The oral microbiota

The oral microbiome is one of the most diverse body niches, only preceded by the colon. The oral cavity is highly diverse due to its many structural and physiological niches harboring a plethora of different microbial communities. These niches include oral mucosa, tongue, saliva, soft tissue, hard tissue, and the surfaces of the teeth. Each surface has distinct communities; hence it provides the conditions and nutrients required for these distinctive microbes. For example, the flora of the tongue differs from that in plaque or the hard tissues of the oral cavity due to its specific microenvironment (Chen et al., 2018). Bacterial and fungal communities play an essential role in the development of many oral diseases such as dental cavities, gingivitis, periodontitis and, subsequently, tooth loss. Bacterial composition consists mainly of Firmicutes, Bacteroidetes, Proteobacteria, Actinobacteria, Spirochaetes, and Fusobacteria (Dewhirst et al., 2010). Among the most dominant bacterial taxa in the oral cavity are *Streptococcus*, *Gemella*, *Abiotrophia*, *Granulicatella*, *Rothia*, *Neisseria*, and *Prevotella* (Dewhirst et al., 2010). The fungi flora is often composed of *Candida*, being the most abundant, *Cladosporium*, *Aureobasidium*, *Saccharomyces*, *Aspergillus*, *Fusarium*, and *Cryptococcus* (Mark Welch et al., 2016). Few studies have analyzed archaeal diversity in the oral cavity; however, methanogenic archaea, like *Methanobrevibacter oralis*, increase in abundance as periodontitis progresses. The oral cavity is sterile before birth (Sulyanto et al., 2019). However, soon after birth, the oral microbiome changes and evolves through adulthood. After 24 h, the newborn oral cavity will most likely be colonized by gram-positive cocci like *Streptococcus* and *Staphylococcus* (Hegde and Munshi, 1998). *Streptococcus salivarius* is an initial colonizer because it is capable of adhering to epithelial cells. From birth to 3 months old, infants have a simple microbial community composed of six main species: *Streptococcus mitis*, *Rothia mucilaginosa*, *Veillonella parvula*, *S. salivarius*, *Gemella haemolysans*, and *Veillonella HB016* (Sulyanto et al., 2019). Between 3 and 6 months old, the infant shows a distinctive microbiota due to solid food ingestion, hygiene, built environment, and contact with other humans and domestic animals; characterized by an increase of *Prevotella*, *Granulicatella*, and *Neisseria* (Sulyanto et al., 2019). The acquisition of these species has been assigned to the emergence of teeth, forming microenvironments, niches, and new surfaces for bacterial colonization and adherence (Kennedy et al., 2019). Late colonizers include *Prevotella*, *Porphyromonas*, *Leptotrichia*, and *Actinomyces,* which colonize infants around 1 year of age (Kennedy et al., 2019). Children with primary dentition have a higher prevalence of *Pseudomonas*, *Acinetobacter*, *Moraxella*, and *Enhydrobacter* (Crielaard et al., 2011). As dentition becomes permanent, populations of *Veillonella* and *Prevotella* increase, while *Granulicatella* decreases (Crielaard et al., 2011). The oral microbiome continues to develop from puberty to adulthood, and lifestyle habits have an impact on microbial diversity. Mucosal surfaces and saliva are primarily composed of aerobic bacteria. However, fissures and supragingival surfaces have a higher abundance of facultative anaerobes, in contrast with the subgingival plaque, which favors strict anaerobes (Arweiler and Netuschil, 2016). During puberty, many hormonal and nutritional changes take place. These changes often lead to an increase of gram-negative anaerobes and spirochetes, which may be associated with a higher incidence and severity of gingivitis. Gingivitis and periodontitis are common bacterial infections that are caused by host immune responses against pathogenic bacteria, leading to inflammation and dysbiosis. While gingivitis is a mild reversible inflammation, if left untreated, it could develop into periodontitis, an irreversible disease that causes chronic inflammation of the gums and subsequent bone loss (Huang et al., 2011). Research conducted on periodontal health and changes after therapy found that plaque samples have more abundance of Fusobacteria, while saliva samples have a higher prevalence of Firmicutes and Proteobacteria, even though the saliva microbiome is likely affected by conditions other than the periodontal disease (Huang et al., 2011). Additionally, Bacteroidetes and Spirochaetes were higher in healthy individuals, while *Porphyromonas*, *Tannerella*, *Prevotella*, and *Filifactor* were more abundant in participants with periodontitis (Huang et al., 2011). Particularly, periodontitis has been associated with a higher risk of oral cancer. Nonetheless, the role of the oral microbiota in the development of oral cancer is not yet well established; however, certain species have been observed at tumor sites (Guerrero-Preston et al., 2016) (Figure 2).

### The microbiota of the gastrointestinal tract: Stomach, intestines, and cecum

The gastrointestinal microbiome is the largest and most diverse reservoir of all the human body niches. From the mouth to the anal cavity, each digestive organ section provides a specific environment that allows the growth and colonization of organisms. The most common phyla across the gut tube are the Firmicutes, Bacteroidetes, Proteobacteria, and Actinobacteria. In the esophagus, the most prevalent bacterial taxa are *Streptococcus*, *Veillonella*, and *Prevotella* (Pei et al., 2004), a composition that resembles that of the oral microbiome (Pei et al., 2004). The microbial communities of the stomach are dominated by Proteobacteria and Firmicutes (Maldonado-Contreras et al., 2011). Many studies have also stipulated that *Helicobacter pylori* is part of the normal flora found in the stomach, which was lost through modern lifestyles (Pei et al., 2004). Other studies found that positive *H. pylori* status was associated with an increased relative abundance of non-*Helicobacter* bacteria from the Proteobacteria, Spirochaetes, and Acidobacteria phyla, alongside a decreased abundance of Actinobacteria, Bacteroidetes, and Firmicutes (Maldonado-Contreras et al., 2011). Despite the fact that *H. pylori* is a causative agent of gastritis and is associated with gastric cancer, other studies demonstrated how *H. pylori* infections could lower the risk of celiac disease (Lebwohl et al., 2013), asthma (Arnold et al., 2011) and esophageal adenocarcinoma (Xie et al., 2013). Gastroesophageal reflux disease (GERD), Barrett’s esophagus, and esophageal carcinoma are all a result of microbial dysbiosis (Pei et al., 2004). Persistent GERD that progresses to Barrett’s esophagus, predisposing to an esophageal carcinoma, has been related to an increase of *Veillonella*, *Fusobacterium*, and *Prevotella*, taxa that are absent in healthy individuals (Liu et al., 2013). The small intestine is characterized by an environment with high concentrations of oxygen and antimicrobials along with a short transit time that allows the rapid growth of facultative anaerobes (Rinninella et al., 2019). It absorbs 90% of the host’s energy from the diet and is divided into three parts: duodenum, jejunum, and ileum. The most abundant phyla in the duodenum are Firmicutes, Proteobacteria, and Actinobacteria, taxa that contribute to most of the nutrient digestion, including protein, lipids, and simple sugars (Angelakis et al., 2015). Particularly, the most dominant genera found are *Prevotella*, *Stenotrophomonas*, *Streptococcus*, *Lactococcus*, *Bacillus*, *Solibacillus*, *Pseudomonas*, *Arthrobacter*, and *Lysinibacillus* (Gong et al., 2019).

In the jejunum, Firmicutes and Proteobacteria are the most predominant, while *E. coli*, *Enterococci*, and *Lactobacillus* were also identified as predominant species of the duodenum and jejunum (Sundin et al., 2017). The ileal microbiota is dominated by *Streptococcus*, *E. coli*, *Clostridium* (Zoetendal et al., 2012); however, with significant inflammation, members of Fusobacteria and Proteobacteria increase significantly with a reduction of Bacteroidetes and Spirochaetes (Fan et al., 2020). The colon, which is the most diverse niche, has an anaerobic environment dominated by Bacteroidetes, especially in the sigmoid colon (James et al., 2020). The most dominant taxa is *Bacteroides*, while *Enterococcus* is more prevalent in the proximal colon, contrary to the distal colon, which has higher abundance of Coprobacilus etc by *Coprobacillus* and *Escherichia*/*Shigella* (James et al., 2020). Key biomarkers of health across the human colon have been identified, including *Lactobacillus, Bifidobacterium and F. prausnitzii* (Khan et al., 2014). Other non-bacterial components of the colon microbiome, which are also important residents, include bacteriophages, fungi such as Ascomycota and Basidiomycota, and archaea such as *Methanobrevibacter smithii* (Hoffmann et al., 2013).

The cecum and appendix sections of the large intestine have a similar composition to those previously described, with a slight reduction in *Bacteroides* (James et al., 2020). The appendix has been characterized by high diversity, with the dominance of Firmicutes, Proteobacteria, Actinobacteria, Bacteroidetes, and Fusobacteria. At the family level, Lachnospiraceae, Enterobacteriaceae, Bacteroidaceae, Fusobacteriaceae, and Bifidobacteriaceae, specifically the genus *Bifidobacterium* were the predominant groups (Guinane et al., 2013). During appendicitis, other non-intestinal genus such as *Fusobacterium Gemella*, or *Parvimonas* have been detected (Guinane et al., 2013). Fecal healthy biomarkers such as *Bacteroides*, *Eubacterium rectale*, *F. prausnitzii*, and *Akkermansia muciniphila* are inversely related with appendicitis (Swidsinski et al., 2011). Despite being considered an organ that has lost its function throughout evolution, the appendix has great biological redundancy ensuring gut repopulation in dysbiotic situations after pathogen colonization, diarrheal diseases, or antibiotic treatments (Guinane et al., 2013) (Table 1).

### Gut microbiota and its implication in obesity and diabetes

Obesity is not just a public health problem for adults, it is estimated that 40 million children worldwide are obese (Chen et al., 2020). This can lead to the future development of T2D, cardiovascular diseases, and some types of cancer. A duodenal microbiota dysbiosis of obese individuals is characterized by an increase in Proteobacteria and a decrease in Firmicutes (i.e., Lachnospiraceae family). In particular, Pseudomonadales increase in obese people compared to lean individuals (Nardelli et al., 2020). Within the roles of the gut microbiota is the maintenance of the energy homeostasis through the fermentation of short-chain fatty acids (SCFAs). Thus, a decrease in Firmicutes, which are SCFAs producers, has been related to reduced protection in the intestinal barrier (Schnorr et al., 2014). A Bacteroidetes to Firmicutes ratio has been used as a barometer for obesity. A meta-analysis of the gut microbiota of diet-induced obese rodents revealed an increase in Firmicutes and Actinobacteria, alongside a reduction of Bacteroidetes in obese compared to lean rodents; with no significant differences in alpha-diversity (Jiao et al., 2018). At the family level, the obese models showed an increase in *Ruminococcaceae* and *Christensenellaceae*, whereas, at the genus level, *Ruminococcus*, *Dorea*, and *Oscillospira* were significantly increased (Jiao et al., 2018). An increase in *Lactococcus* was associated with higher inflammation in obese individuals. Furthermore, obese children have a greater abundance of *Campylobacter, Actinobacillus*, *Aggregatibacter*, *Streptococcus*, and *Rothia* (Chen et al., 2020). This microbial dysbiosis can have serious consequences such as an abnormal absorption of polysaccharides and proteins as well as disrupted pathways such as cellular processes, genetic information processing, and metabolic disturbance (Chen et al., 2020). In addition, obesity-associated dysbiosis can lead to the poor fermentation process of polysaccharides and bile acid dihydroxylation (Jiao et al., 2018).

**FIGURE 3:**
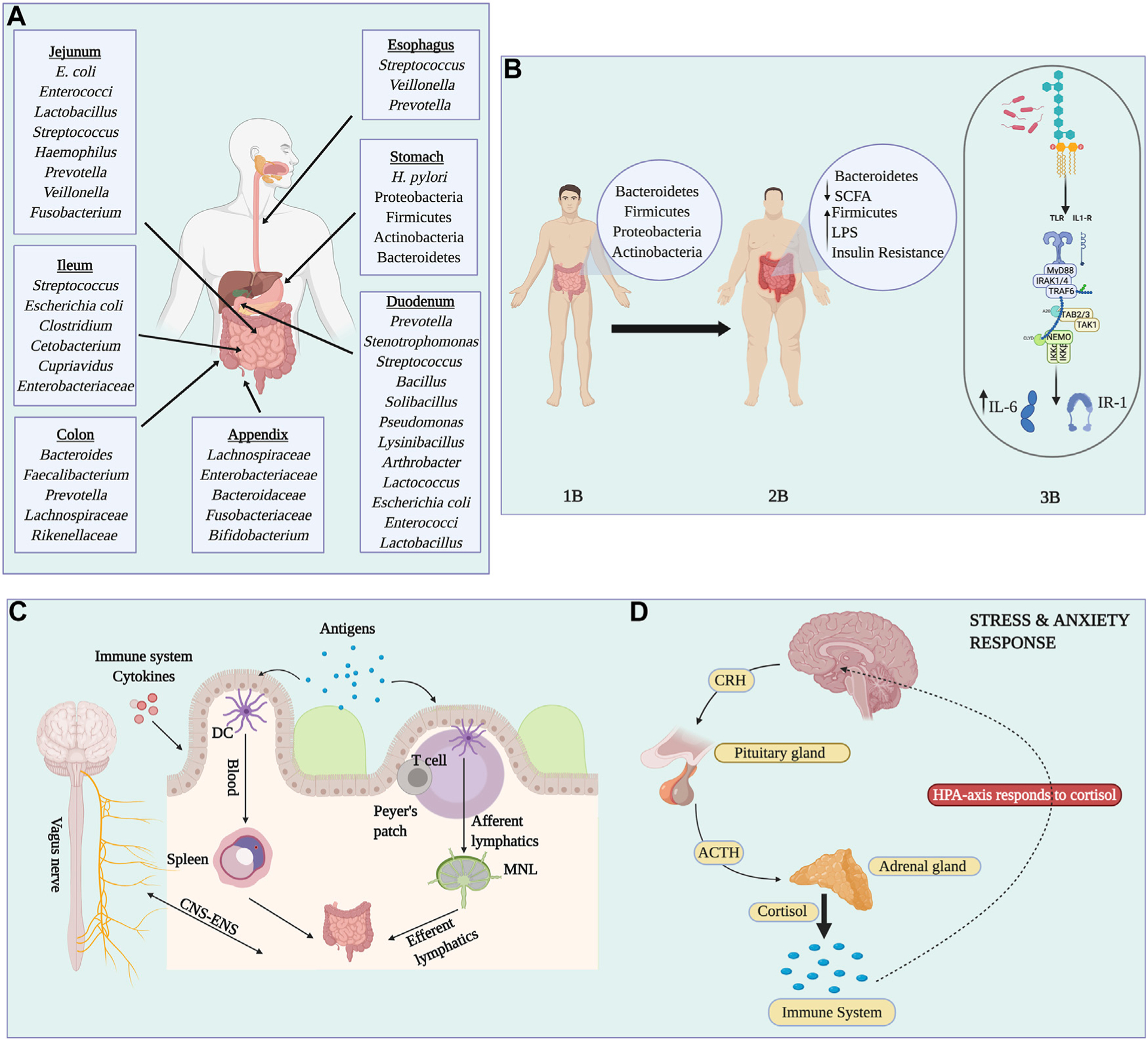
Complexity of the human gut microbiota. Panel **(A)** Representation of the most characteristic and predominant organisms found in each gastrointestinal site. Panel **(B)** Representation of the differences between individuals with a healthy lifestyle compared to obese individuals. Normal/lean microbiota are dominated by Bacteroidetes, Firmicutes, Proteobacteria, and Actinobacteria (1B). Obesity, diabetes, and gut inflammation are characterized by an increase in Firmicutes and Lipopolysaccharides (LPS), and a reduction in Bacteroidetes and short-chain fatty acids (2B). The production of LPS is recognized by Toll-Like receptor four which produces inflammatory interleukins (IL-6) and phosphorylation of Insulin Receptor-1 which is associated with insulin resistance (3B). Panel **(C)** Represents how bacterial antigens enter the Peyer’s patches through M cells and are captured by dendritic cells (DCs). Lymphocytes interact with antigen-loaded DCs and they migrate to the lymph nodes causing expansion, differentiation and proliferation. In addition, antigens can be transported to the spleen by circulation. Antigens are processed and presented to T cells, initiating an immune response. Finally, effector T cells return to the gut lamina propria where they reside. Panel **(D)** shows how alterations in gut microbiota can cause stress and anxiety-like disorders. Image created with BioRender.com.

These metabolic disturbances can have far-reaching consequences, including the development of T2D (Gaike et al., 2020). A reduction of *Akkermansia* and *Blautia* was found in diabetic individuals. These also have lower levels of butyrate-producing bacteria such as *Roseburia intestinalis* and *F. prausnitzii,* alongside elevated amounts of *Lactobacillus gasseri* and *Streptococcus mutans* (Pitocco et al., 2020). Diabetes and obesity have been implicated with insulin resistance because of the inflammation related to the lipopolysaccharide (LPS) metabolites secreted by bacteria. In particular, LPS secreted by Firmicutes is increased in obese and diabetic individuals (Saad et al., 2016). These LPS are detected by Toll-like receptor four (TLR-4), which activates a signaling pathway that leads to the secretion of inflammatory cytokines such as IL-6 and Interferon-alpha (Saad et al., 2016). In addition, serine kinases are also activated, which act as substrates for insulin receptors, thus, promoting insulin resistance (Saad et al., 2016). *Akkermansia muciniphila*, a very dominant gut bacteria, has proved to be inversely correlated with body weight, adiposity, blood glucose, and intestinal permeability. In fact, the administration of *A. muciniphila* to mice on a high-fat diet resulted in an increase in the mucus layer as well as the restoration of tight junction proteins, antimicrobial peptides, and anti-inflammatory bioactive lipids; and was linked to lower adiposity and low insulin resistance (Lecerf and Cani, 2022). A summary of body niche bacteria, gut bacteria between lean and obese individuals, and the details of the gut immune trafficking are found in Figure 3.

### Role of the gut microbiota in metabolizing drugs and modulating immunotherapy against cancer

The gut microbiome is also involved in metabolizing drugs and is related to therapy efficiency. A study by Zimmermann et al. examined how 76 different gut microorganisms could have enzymes that metabolize and chemically modify 271 oral drugs (Zimmermann et al., 2019). These findings showed how some drugs cause more severe side effects in some individuals than others, mainly because of the interpersonal microbiome variations (Zimmermann et al., 2019). In another study on the inactivation of digoxin- a drug to treat heart failure and arrhythmias- researchers found that *Eggerthella lenta* reduced digoxin potentially by using it as an alternative electron acceptor, which leads to a decreased target affinity of the drug (Haiser et al., 2013). Similarly, anti-cancer drugs can be modified by the gut microbiota. Irinotecan, an anti-cancer drug used for a range of solid tumors (Wallace et al., 2010), is inactivated through the addition of glucuronic acid (GlcA). However, the bacterial protein β-glucuronidase (GUS) removes GlcA and reactivates irinotecan, which leads to epithelial damage and diarrhea (Wallace et al., 2010; Bhatt et al., 2020). Recent studies have targeted the GUS protein with inhibitors which effectively prevented intestinal toxicity (Wallace et al., 2010), overgrowth of *Enterobacteriaceae*, and maintained the antitumor effect of irinotecan (Bhatt et al., 2020). Immunotherapy has risen as a more sought treatment when dealing with cancer, mainly because of the complex interactions that occur between a patient’s immune system and the tumor. Particularly, immune checkpoint inhibitors targeting CTLA-4 and PD-1 have been developed; however, variable responses to these treatments have been associated with the gastrointestinal (GIT) microbiome. A recent study aimed at understanding the role of the gut microbiome in response to immune checkpoint inhibitors targeting PD-1 in patients with metastatic melanoma, showed that responder patients had a higher alpha diversity and relative abundance of Clostridiales, *Ruminococcaceae*, and *Faecalibacterium* when compared to non-responders (Gopalakrishnan et al., 2018). In addition, an oncolytic adenovirus efficacy against malignant glioma in mice, seems to be also modulated by gut bacteria, with an increase in Bifidobacteria and Lactobacilli associated with a better response to the therapy(REF: https://aacrjournals.org/cancerres/article/81/13_Supplement/927/669706).

### An overview of the gut-brain axis

The gut-brain is a bidirectional link between the central nervous system (CNS) and the enteric nervous system (ENS), which communicates between four information carriers in the so-called gut connectome: 1) the vagal and spinal afferent neurons, 2) immune messages carried by cytokines, 3) endocrine messages carried by gut hormones and 4) microbial factors that reach the brain through the bloodstream (Holzer and Farzi, 2014; Boem and Amedei, 2019). The communication between these carriers is important for metabolic activities and for maintaining microbial homeostasis, as some gut hormones play an important role in the activation of afferent neurons and the vagus nerve (Ye and Liddle, 2017). In the GIT system, the microbiota controls the enteric neurons and motility through transmitters like SCFAs (Obata and Pachnis, 2016), 5-hydroxytryptamine (5-HT, serotonin) (Obata and Pachnis, 2016), γ-aminobutyric acid (GABA) (Pokusaeva et al., 2017), hormones such as cortisol (Valles-Colomer et al., 2019) and immune system modulators such as quinolinic acid (Valles-Colomer et al., 2019). Diseases like schizophrenia and autism have been associated with alterations in gut permeability and even though research has expanded in the last few years, there is still a lack of information on some groups, such as Hispanics (Vera-Urbina et al., 2022).

Autism spectrum disorder (ASD) is a neurodevelopmental disorder that has been linked to changes in gut microbiota. The microbiota of healthy children was shown to be composed of Bacteroidetes, Firmicutes, and Actinobacteria with a higher abundance of *Coprococcus* and *Bifidobacterium* (Iglesias-Vázquez et al., 2020). Children who suffer from ASD have a greater composition of *Bacteroides*, *Parabacteroides*, and *Clostridium*, and a lower abundance of *Coprococcus* and *Bifidobacterium* (Iglesias-Vázquez et al., 2020). In addition, research shows that *Clostridium* releases toxins that can affect the brain. Alterations in SCFA production can affect homeostasis and increase inflammation. SCFAs are speculated to regulate neuro-Immuno endocrine functions as some are able to cross the blood-brain barrier and even maintain barrier integrity, thus helping control the passage of molecules and nutrients from the circulation to the brain (Silva et al., 2020). ASD patients have demonstrated a lower abundance of *Bifidobacterium*, which plays a role in producing Gamma-Aminobutyric Acid (GABA)—a natural brain neurotransmitter—and ultimately translating into cognitive deficits (Garcia-Gutierrez et al., 2020). Lower levels of GABA affect glutamate metabolism, which leads to anxiety and behavioral disorders. Abundance in taxa such as *Escherichia*, *Bacillus* or *Saccharomyces* can produce noradrenaline (Barrett et al., 2012), affecting its uptake by the hypothalamic-pituitary-adrenal (HPA) axis, which centralizes the stress response system (Scriven et al., 2018). Other studies have revealed alterations in tryptophan metabolism with a concomitant increase in serotonin, affecting different behaviors such as sleep, appetite, emotions, and social skills (Roth et al., 2021). Moreover, treatment with propionic acid (PPA) in an animal model of autism showed enhanced inflammation with an increase in pro-inflammatory cytokines like IL-6 and TNF-α (Aabed et al., 2019).

Other neuropsychiatric diseases like depressive disorders are associated with gut dysbiosis. It has been demonstrated that transplantation of gut microbiota from depressed mice to germ-free mice results in the display of depressive behavior (Knudsen et al., 2021). Anxiety and depression have also contributed to the global obesity burden, and authors suggest a role of gut-brain axis malfunction through disruptions in the crosstalk between the immune and the endocrine systems (Niccolai et al., 2019).

A decrease in Firmicutes accounts for a decline in SCFAs with depression, affecting the intestinal barrier (Huang et al., 2018). A study showed that women with depressive disorders had lower concentrations of SCFAs in contrast to non-depressive women (Capuco et al., 2020). The significant decrease in Firmicutes associated with depression includes a decline in *Akkermansia*, *Ruminococcaceae*, and *Dorea*; with a simultaneous increase in Actinobacteria*, Prevotella*, and *Parabacteroides* (McGaughey et al., 2019). Bifidobacteria levels were also reduced in depression, nonetheless the restoration of some species like *Bifidobacterium longum* and *B. breve* reduced depressive behaviors and increased the secretion of 5-hydroxytryptophan and butyrate (Tian et al., 2019).

Stress is a major disruptor of gut homeostasis. It can alter the intestinal barrier, increase gut permeability dysfunction and induce changes in the HPA-axis. Stress can affect gut microbial composition stimulating inflammatory mechanisms due to the release of pro-inflammatory cytokines. An element responsible for cytokine release is NF-kB, a transcription factor that, when inhibited by *Bifidobacterium adolescentis* results in a positive effect on stress-related diseases (Guo et al., 2019). Additionally, people with anxiety disorders have a decrease in *Lactobacillus rhamnosus* (Slykerman et al., 2017). Prenatal stress causes changes in the brain and behavior that can contribute to the development of gastrointestinal and psychiatric disorders. An animal study showed how supplementation of Lactobacilli could reverse anxiety behavior in stressed rats accompanied by normalized levels of adrenocorticotropic hormone (ACTH) and corticosterone (Karen et al., 2021).

Another major disorder of the gut-brain axis is Alzheimer’s Disease (A.D.), which is associated with memory deficit due to the accumulation of β-amyloid plaques (Aβ) (Nimgampalle and Kuna, 2017). Apolipoprotein E (ApoE) is the major risk for developing A.D. because it enhances the production of Aβ. The synthesis of ApoE is induced by neurons and is stimulated by stressors like age, oxidative stress, or trauma (Mahley et al., 2009). Microbial dysbiosis leads to an increase in microbial-associated chemicals, like LPS, which play a vital role in activating innate immunity and triggering neuroinflammatory pathways in the brain’s microglia. Acetate a SCFA promotes microglia development and can slow disease progression (Erny et al., 2021). Patients with A.D. present an increased proportion of pro-inflammatory taxa in the intestines, and altered gut microbiota enhances cerebral aggregation and deposition of Aβ plaques by immune, endocrine, and neural pathways (Dumitrescu et al., 2018). A decrease in butyrate-producing bacteria such as *Butyrivibrio* (*B. hungatei and B. proteoclasticus*), *Clostridium* sp. *strain SY8519*, *Eubacterium* (*E. eligens*, *E. hallii*, and *E. rectale*), *F. prausnitzii* and *Roseburia hominis* is also observed in A.D. patients (Haran et al., 2019).

### Degradation of the gut microbiome: Urbanization and westernized lifestyles

**FIGURE 4:**
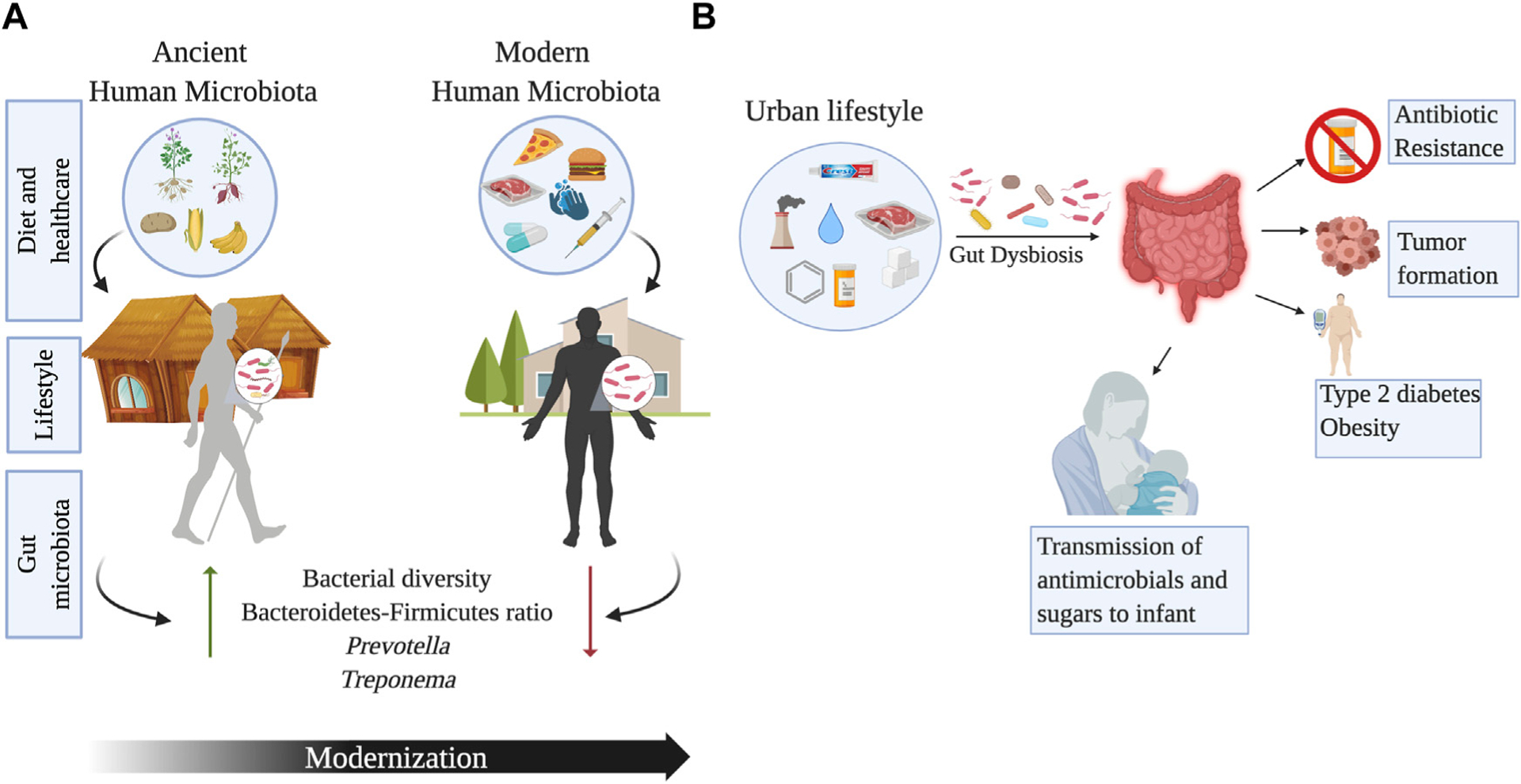
Changes in the gut microbiome across urbanization and human lifestyles. Panel **(A)** shows the impact of modernization on the gut microbial diversity due to changes in diet, healthcare, sanitation, and lifestyle associated to modernity. Ancient microbiomes have higher microbial diversity and may even include taxa such as *Treponema*, that is no longer a component of the modern human microbiota. Panel **(B)** outlines the effects of urbanization on the gut microbiome. Increase in the consumption of refined sugars, antibiotics, chemical antimicrobials, exposure to air pollution and water chlorination, can have detrimental effects on the gut microbiota leading to the development of metabolic conditions, colorectal cancer, and antibiotic resistance. Image created with BioRender.com.

The “Missing Microbe” hypothesis postulates that industrialization and current medical practices (e.g., vaccination) have diminished the prevalence of infectious diseases such as tuberculosis and malaria (Blaser and Falkow, 2009). However, access to healthcare and improved life expectancy has reduced gut microbial diversity. Urbanization is the main cause of major human microbiome shifts and microbial loss, as documented by studies evaluating rural and urban lifestyles (Lokmer et al., 2020) (Figure 4). The transition of diet and lifestyles has impacted the human gut microbiome, from changes established during the hunter-gatherer transition to agriculture to our modern time transition to urbanization (Jha et al., 2018). For instance, the BaAka hunter-gatherers of the Central African Republic have a high relative abundance of *Anaerovibrio*, *Sutterella*, and unclassified members of *Clostridiaceae* and Cyanobacteria (Schnorr et al., 2014). By contrast, their agriculturalist neighbors, the Bantu, have a higher abundance of *Christenellaceae*, *Dialister*, *Faecalibacterium*, *Lactococcus*, *Leuconostoc*, *Mogibacteriaceae*, and *Ruminococcaceae* (Schnorr et al., 2014). However, no differences in bacterial diversity were observed between these groups (Gomez et al., 2016).

The Yanomami, indigenous peoples of the Amazonian jungle in Venezuela, have the most diverse gut-bacterial biota ever found in humans and a significant increase in diversity compared to people in the United States (Clemente et al., 2015). This semi-nomadic tribe lives in isolation in southern Venezuela, maintaining ancestral lifestyles (Clemente et al., 2015). The Yanomami fecal microbiota was characterized by high levels of *Prevotella* and low *Bacteroides*, contrary to what was observed in the United States. Indeed, the study by Dominguez-Bello highlights a significant difference in beta-diversity among indigenous peoples and samples collected from Americans (Clemente et al., 2015). The frequent meals and food seasonality in the Yanomami contrasts with the large, infrequent meals characteristic of Western diets. Paradoxically, the study found no significant differences in oral bacteria, possibly explained by chewing of tobacco in Amerindians from an early age (Clemente et al., 2015). Chinese urban populations have also shown a decrease in diversity at all phylogenetic levels, including a loss of Archaea and viruses compared to rural samples. Urbanization in these groups was also associated with a higher number of virulence and antibiotic resistance genes (Winglee et al., 2017). A study in Thailand comparing the gut microbiota of children living in urban Bangkok (whose diet consisted of high sugar, fats, and protein) with children living in rural Buriram (with a diet rich in vegetables and rice) found a loss of diversity and lower levels of SCFAs in urban samples. Additionally, rural samples had a higher prevalence of *Ruminococcaceae* and enriched gene profiles involved in plant metabolism (Kisuse et al., 2018). Similar to urban North Americans and Asians, the microbial communities of children living in urban Burkina Faso and Italy differed from those of children living in rural Burkina Faso (De Filippo et al., 2017). Rural children contained gut fiber-degrading bacteria (*Prevotella*, *Treponema*, and *Succinivibrio*), whereas children living in urban areas had bacteria that metabolize animal protein (De Filippo et al., 2017). One key aspect of living in an industrial/urban setting is the availability of drinkable water and the process of water purification (chlorination). Some studies have shown that chlorine concentrations in water can promote the incidence of colon cancer (Benmarhnia et al., 2018). Stool samples of mice subjected to chlorine water have seriously reduced *C. perfringens* and moderately reduced *C. difficile*, *Enterobacteriaceae*, and *Staphylococcus* (Sasada et al., 2015). The use of cosmetics and cleaning products in urbanized areas can also be linked to changes in gut microbiota. Triclosan (TCS) is a chemical well known to be present in toothpaste as it possesses antimicrobial properties. The gut microbiota of infants-mother dyads exposed to TCS has shown an enrichment in Proteobacteria, which serves as a marker for antibiotic resistance in the gut microbial community (Ribado et al., 2017). On the other hand, individuals using non-TCS containing toothpaste showed enrichment of *B. fragilis* which is linked to the production of anti-inflammatory polysaccharides. The use and exposure to TCS has also been correlated with an impact in the gut microbiome of infants through breast milk (Bever et al., 2018). The presence of TCS in the breast milk of mothers who use personal care products can affect the microbial diversity of infants at an early stage of gut microbial community development, thus paving the way for future health problems. Another disrupter of intestinal eubiosis can be air pollutants. Young adults living in Southern California exposed to high levels of ozone showed lower microbial diversity, along with higher levels of *B. caecimuris* (Fouladi et al., 2020). Furthermore, higher exposure to nitrogen dioxide correlated with higher Firmicutes and less diverse taxa (Fouladi et al., 2020). Interestingly, the exposure to traffic-related air pollution has been shown to alter the abundance of *Bacteroidaceae* and *Corynebacteriaceae*, which have been linked to obesity (Alderete et al., 2018). Modern food processing has led to the loss of microbial diversity compared to rural environments (Obregon-Tito et al., 2015). Non-thermal and thermal processed food can affect the gut microbiota of vertebrates, as seen in mice and catfish. Vertebrates with thermal processing food diets had less diversity and a different microbial composition according to the host (Zhang and Li, 2018). Dietary toxic xenobiotics such as nitrosamines, polycyclic hydrocarbons, and heterocyclic amines in processed foods are also associated with a change in the gut microbiota, increasing the risks of developing colorectal cancer (Zhang and Li, 2018). A murine model exposed to the environmental pollutant benzo(a)pyrene showed an increase in *Bacteroidaceae*, *Paraprevotellaceae*, and *Porphyromonadaeae* alongside inflammation of colonic mucosa, as well as a decrease in *Lactobacillaceae* and *Verrucomicrobiaceae* (Ribière et al., 2016). Preservatives, from salt to chemical components, have been used in urban countries for long-term food storage. Some of these preservatives, like emulsifiers, may alter the gut microbiota (Chassaing et al., 2017). Additionally, prolonged exposure to Saccharin causes gut microbiota dysbiosis and inflammation in mice, as shown by an increase in *Corynebacterium* spp. with a reduction of key anti-inflammatory taxa, including *Dorea* and *Ruminococcus* (Bian et al., 2017). Saccharin is also related to a decrease in Equol production. Equol is formed by gut bacteria during the metabolization of daidzein (Isoflavone), a factor contained in soy products. Even though equol is known for having antioxidant and anti-carcinogenic properties (Bian et al., 2017), extensive consumption of both equol of dairy products in Hispanics was associated with reduced gut health (Lacourt-Ventura et al., 2021).

### Extinct populations—What do archeological findings tell us?

The evolution of the human microbiota can be addressed from a paleontological point of view using ancient microbial DNA, preserved in archeological samples. Human coprolites (mummified feces) collected from Cueva de Los Chiquitos, Rio Zape Valley, Mexico dating 1,300 years B.P., were similar to each other and to feces from rural populations with higher levels of *Prevotella* and *Treponema* (Tito et al., 2012). These bacteria are commonly found in traditional communities and are rare or missing in modern gut microbiomes (Tito et al., 2012). Similarly, the gut microbiome preserved in coprolites from the United States and Mexico were more similar to rural gut microbiomes than to industrial ones (Wibowo et al., 2021). In particular, coprolites and extant non-industrial fecal samples have a higher prevalence of *Ruminococcus callidus*, *Butyrivibrio crossotus* and *Treponema succinifaciens,* whereas industrial fecal samples had a higher abundance of Bacteroidetes such as *Bacteroides* and *Prevotella* (Wibowo et al., 2021). Recently, paleomicrobiological studies showed that coprolites from two ancient agricultural ethnic groups (Huecoid and Saladoid) from Puerto Rico, exhibited microbial community differences due to diet-related to cultural traditions (Cano et al., 2014). Maize and Basidiomycetes sequences were found in Huecoid coprolites, whereas sequences related to fish parasites were detected in Saladoid, suggesting the consumption of maize and fish, respectively (Cano et al., 2014). Similar to that observed in coprolites, the gut microbiome of Peruvian Inca mummies differs from that of modern Amazonians (Santiago-Rodriguez et al., 2016). Analyses of the 16S rRNA gene showed that Pseudomonadales and Enterobacteriales were more abundant in modern Amazonians, whereas Lactobacillales were more abundant in the Peruvian Inca mummies. In addition, *Trypanosoma cruzi* sequences were detected in higher abundance in mummies compared to modern Amazonians suggesting the presence of Chagas disease in South America before the arrival of Europeans (Santiago-Rodriguez et al., 2016). Overall, ancient DNA studies could be essential to understanding the changes in the human microbiome and the evolution of pathogens. However, with ancient samples, a major caveat is DNA degradation, which may favor the amplification of modern DNA due to contamination. Furthermore, sequencing errors may produce distorted DNA sequences that could lead to bias in taxonomic assignments. Therefore, hunter-gatherers and agriculturalists are better suited for the study of ancient microbiomes (Figure 4).

### Microbial restoration—A potent new avenue in microbiome research

**FIGURE 5:**
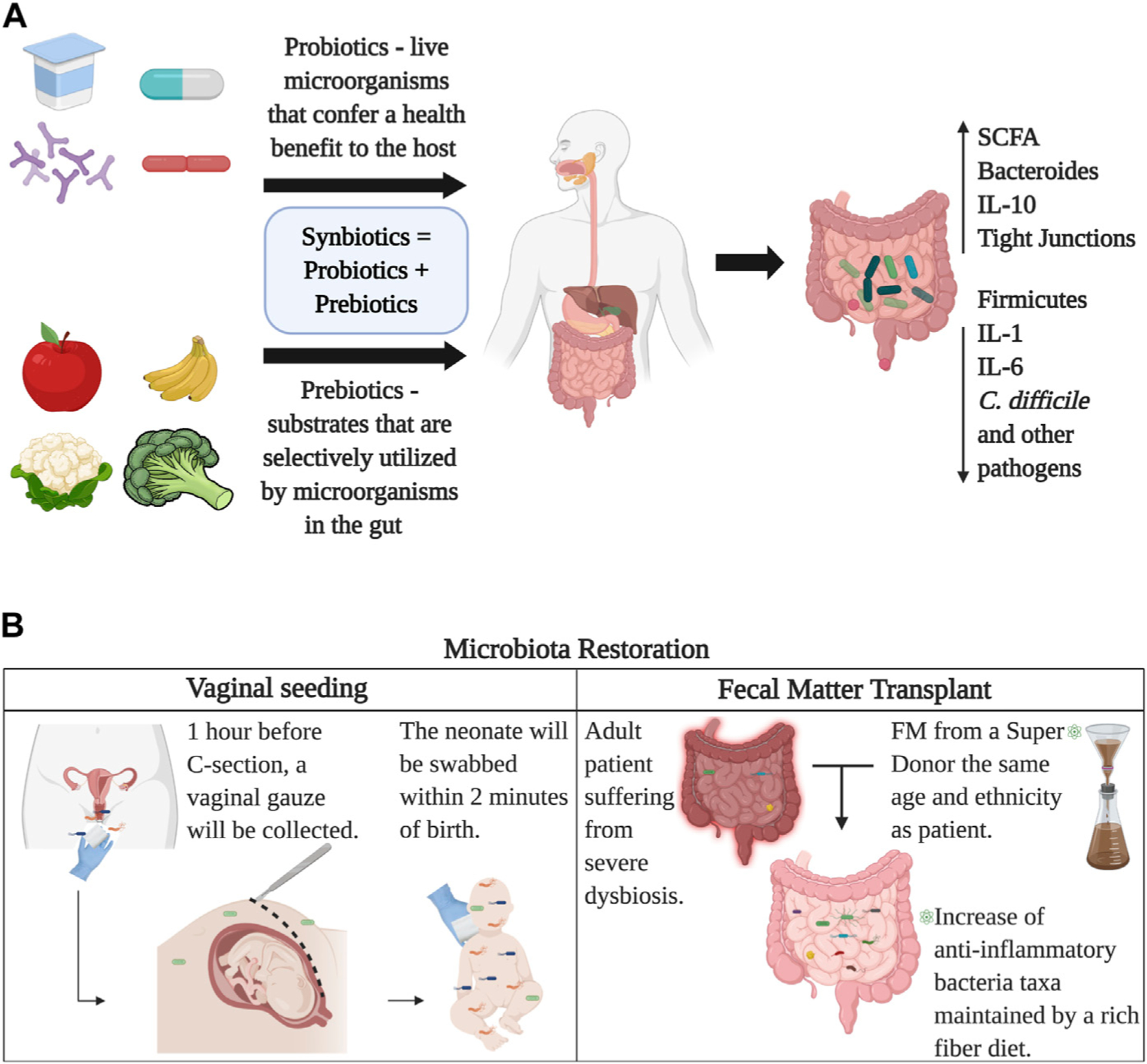
Restoration of the gut microbiome. Panel **(A)** Probiotics provide live microorganisms in the form of food or supplements that directly colonize the gut, while prebiotics provide fiber and carbohydrates that stimulate the growth of healthy bacterial colonies that already reside in the gut. Together, probiotics and prebiotics compose synbiotics which can be obtained commercially. Panel **(B)** displays two ways of microbial seeding, vaginal transfer for C-section born babies and fecal matter transplants. Image created with BioRender.com.

Diet and nutrition are known as the best modifiers of the gut microbiota. Specific nutrients are known to modulate the amounts and types of gut bacteria, as explained before. For instance, protein metabolism from beef induces a lower abundance of *Bifidobacterium adolescentis* and higher prevalence of *Bacteroides* and *Clostridia* when compared to consumers of a meatless diet (Singh et al., 2017). Proteins from whey and peas, however, increase B*ifidobacterium* and *Lactobacillus*, who function as anti-inflammatory taxa and SCFA-producers and decrease *Clostridium perfringens* (Singh et al., 2017). Consumption of saturated and trans fats is associated with cardiovascular disease risk (Estadella et al., 2013). Studies have suggested that a high-fat diet increases total anaerobic microflora and counts of *Bacteroides*, produces a lower concentration of *Lactobacillus* and is also related to inflammation and insulin resistance (Wu et al., 2011). A study found that a low fat, high carbohydrate diet increased fecal *Bifidobacterium*, and reduced fasting glucose and cholesterol when compared to baseline (Fava et al., 2013). A high unsaturated fat diet leads to an increase in lactic acid bacteria and *Bifidobacteria*. Saturated fat, however, promotes the proliferation of *F. prausnitzii*, *Bilophila*, and *Bacteroides* (Singh et al., 2017). As for carbohydrates, these are either digestible or non-digestible. Digestible carbohydrates, including starches and sugars, are degraded by digestive enzymes in the small intestine (Seo et al., 2020). On the contrary, non-digestible carbohydrates include fiber (cellulose and hemicellulose) and resistant starch, which can only be digested by fermentation through resident microorganisms of the large intestine (Sonnenburg and Sonnenburg, 2014). The fermentation of fiber changes the composition of the gut microbiota favoring bacteria capable of these processes. Fibers are thereby designated as prebiotics. A diet based on vegetables and fruits can act as prebiotics, providing polyphenols (Henning et al., 2017) and reducing the levels of Firmicutes and increasing *Bacteroides* (Henning et al., 2017). It can also lead to an increase in the abundance of *Akkermansia* and *Bifidobacteria*. The American Gut Project—the biggest microbiome citizen science project to date—has revealed that one does not need to be vegan or a strict vegetarian to have good gut bacteria, the important thing is to consume a variety of plants per week. It’s been documented that individuals who consume more than 30 types of plants per week compared to those who consume ten or fewer, had significantly reduced abundance of antibiotic resistance genes and gut diversity (McDonald et al., 2018). In summary, a balanced diet can improve gut microbiota, metabolic functions, and overall health (Figure 5A).

Synbiotics are products that have both probiotic and prebiotic components (Anand et al., 2019). An example is the use of *Lactobacillus* and *Bifidobacterium* along with carbohydrates (Rigo-Adrover et al., 2018). The use of synbiotics, although new, has given promising results. In a double-blind placebo-control trial, administration of a synbiotic reduced neonatal death and sepsis between the placebo group and treatment group by 40% (Panigrahi et al., 2017). In addition, to help maintain homeostasis in the gut and restore normal microbial populations, probiotics, prebiotics, and synbiotics have shown they can serve as alternative treatments for different conditions, as well as boost body functions. Fermented foods can either be fermented naturally -microorganisms pre-exist in raw food, or can be fermented using starter cultures, i.e., adding microbial colonies to already existing food products (Rezac et al., 2018). Historically, fermented foods have stood out for their long shelf life, but in recent years they have gained popularity because of their potential health benefits, especially gut health, since these fermented foods contain probiotic bacteria and prebiotic components. One of the most widely known fermented foods that have proven to have a beneficial effect on the gut microbiota is yogurt (Le Roy et al., 2022). More specifically, recent studies found that the use of fermented yogurt with probiotic *Lactobacillus* improves blood glucose and insulin levels in rats with T2D and increases the production of SCFAs (Qu et al., 2018). Cheese is another food that has been widely consumed around the world for centuries. A recent study found beneficial effects in cream cheese containing *Lactococcus chungangensis*. Specifically, it was found that rats that were administered cream cheese containing this microorganism presented lower IgE levels, increased fecal *Bacteroides, Lactobacillus* and *Ruminococcus*, as well as increased SCFA production (Kim et al., 2019b).

Additionally, there has been a recent interest in how prebiotic compounds in fermented foods benefit gut diversity and overall health. A study with fermented milk containing probiotic *Bifidobacterium breve* and prebiotic galactooligosaccharides found that people who were administered this milk had increased hydration and defecation as well as more clear skin (Mori et al., 2016); suggesting the potential beneficial effects on skin and gut health that a combination of probiotics and prebiotics can have, i.e., synbiotics (Figure 5A). In addition to traditional fermented foods that are consumed around the world, such as milk, cheese, and yogurt, there are three fermented foods that have proven to have a beneficial effect on gut and overall health: Kefir, Kimchi, and Kombucha. Kefir is a fermented milk drink with a sour taste that is produced using a starter culture that contains yeasts such as *Kluyveromyces* and *Saccharomyces* as well as bacteria such as *Lactobacillus, Leuconostoc*, and *Acetobacter* (Prado et al., 2015). Indeed, Kefir has been shown to alleviate obesity and hepatic steatosis in high-fat diet-fed mice by modulation of gut microbiota and mycobiota (Kim et al., 2017). Lactulose, a synthetic sugar added to Kefir has also been found to have prebiotic effects by increasing Bifidobacteria and defecation (Sakai et al., 2019). Thus, Kefir can increase gut diversity and benefit health by providing both probiotics and prebiotics. Kimchi is a group of fermented and salted vegetables that include cabbage, carrot, pear, apple, and ingredients such as chili, pepper, soybean, and ginger (Patra et al., 2016). Kimchi is usually a fermented food that has high concentrations of *Leuconostoc*, but can also have good concentrations of *Lactobacillus, Weissella,* and *Pseudomonas* (Jeong et al., 2013). Apart from bacteria, Kimchi has also been found to contain archaea and yeast (Chang et al., 2008). A study found that *Lactobacillus Plantarum,* which is usually found on Kimchi, can reduce mesenteric adipose tissue and increase the genomic expression of lipid oxidation genes (Park et al., 2017), features that suggest Kimchi may contribute to weight loss. Originating in China, Kombucha is a popular fermented tea beverage that is produced through aerobic fermentation and uses both bacteria and yeast (Gaggìa et al., 2018). Acetic acid and lactic acid-producing species such as *Acetobacter* and *Lactobacillus*, as well as yeasts such as *Saccharomyces* are the most commonly found organisms in Kombucha (Coton et al., 2017). The acid produced by these bacteria lower the pH of Kombucha, making it difficult for pathogenic bacteria such as *E. coli* to grow inside the beverage (Gaggìa et al., 2018). Although only recently Kombucha has been associated with benefits in health and the microbiota, a recent study found that administration of Kombucha tea reduces fat accumulation in the liver of rats with non-alcoholic fatty liver disease and decreases specific bacteria such as *Clostridium* in a mouse model (Jung et al., 2019). A study found that *Gluconobacter*, a bacteria commonly found in Kombucha, can produce D-Saccharic acid-1,4-lactone - a compound that has been this compound has been found to inhibit oxidative stress and the release of pro-inflammatory cytokines (Bhattacharya et al., 2013).

Microbial communities can also be restored by microbial seeding and fecal matter transplants (FMT). Even though microbial dysbiosis is expected as aging occurs and depends on environmental and birth-related factors, several restoration techniques have been developed to regulate microbial communities and improve health. So far, the best examples of microbiome restoration are vaginal and fecal microbial transfer to neonates (seeding) and fecal microbiota transplantations. The vaginal microbial transfer has been recently reported as the technique of acquiring vaginal bacterial communities from a mother awaiting a C-section and smearing the baby after birth (Dominguez-Bello et al., 2016).

The target of the vaginal seeding is to partially restore the microbiome of a baby born *via* C-section, using the vaginal microbiota of the mother upon delivery (Dominguez-Bello et al., 2016). The purpose is to transfer the vaginal flora from mothers to the mouth, nose, and skin of the newborn. Nonetheless, restoration of the neonatal microbiota born *via* C-section with maternal vaginal microbes has raised concerns about infection risks (Dominguez-Bello et al., 2019). Not all mothers qualify as candidates to be donors of vaginal fluids for the seeding procedure. Only women who were not carriers of infectious diseases nor tested positive for Sexually Transmitted Diseases (STDs) at the time of delivery were allowed to donate (Dominguez-Bello et al., 2016). Also, oral-fecal transplantation has proven successful in changing the neonate microbiota. De Vos and colleagues theorized that vaginally born babies might get their microbes from accidentally ingesting their mother’s stool during the birthing process (Helve et al., 2021). They diluted fecal matter into breast milk donated from the bank and pumped from the mothers themselves—and then fed it to their babies. The gut microbiome of the babies later resembled those born vaginally (Helve et al., 2021).

FMT is the administration of fecal matter from a healthy adult donor into the intestinal tract of an affected adult to change and restore their microbiome to healthy conditions (Pigneur and Sokol, 2016). In recent studies, it has been recognized that FMT is extremely efficient in treating inflammatory bowel disease (Tanaka and Nakayama, 2017), psoriasis (Yin et al., 2019), Crohn’s Disease (Sarrabayrouse et al., 2020), *Clostridium difficile* infections (Cammarota et al., 2017), and ulcerative colitis (Lleal et al., 2019). In addition, FMT has been known to suppress intestinal apoptosis, which reduces inflammatory responses, regulates lymphocytes, and alters the microbiota (Burrello et al., 2019). It has been shown that fecal matter from a healthy donor has the capacity to overturn dysbiosis by restoring alpha-diversity and increasing the abundance of health-related microbiota (Lleal et al., 2019). The long-lasting effects of FMT have been reported in patients with Irritable Bowel Syndrome (IBS) (El-Salhy et al., 2022). It has been reported that both recipient and donor bacterial strains in the FMT recipient persist after 3 months of the procedure, leading to a positive result against previous inflammatory disorders (Li et al., 2016). Lastly, cytokine testing is now performed to assess the accurate reduction of inflammation within the intestines by analyzing the recipient’s fecal matter with mucosal biopsies (Burrello et al., 2019). FMT has the capability to control chronic intestinal colitis by instigating a cascade of anti-inflammatory immune regulations, thereby supporting its usage in individuals with severe intestinal dysbiosis (Burrello et al., 2019) (Figure 5B).

### Long term storage of microbiomes: The Microbiota Vault project

Currently, the global diversity of the human-associated microbiota is threatened by the westernization of lifestyles in the context of urbanization and the shrinking of indigenous cultures, in which a much higher microbial diversity has been observed. While the scientific discovery of causal relationships between individual microbes or microbial communities and human health is still in its infancy, means to protect and preserve microbial diversity may become critical to conserve long-term human health. Metagenomic analyses of the human microbiome revealed that ~80% of the bacteria inhabiting the human body are unknown, prompting the metaphor of “microbial dark matter” (Eckburg et al., 2005). Such unknown diversity also extends to archaea, microbial eukaryotes, and viruses. Taken together, this means that there is a danger of irrevocably losing valuable information and opportunity at a time when science has just started understanding the health relevance and potential of our microbial environment and the microbiome. Hence the need for a global collection of such microbiota and for their safe storage and preservation. Within the Microbiota Vault initiative, a pioneer team of international experts has come together with the aim of safeguarding microbial diversity by supporting collection efforts and creating an institution for safe preservation, the Microbiota Vault. The initiative takes inspiration from the Svalbard Global Seed Vault, which safeguards the global diversity of food crop seeds. The program is supported by several nonprofit foundations and academic institutions, which published a feasibility study in order to assess and concretize the concept for its implementation. This study found that the Microbiota Vault initiative has great significance and potential and urged its leaders to establish a pilot project that would include infrastructure to store diverse microbiomes of the world’s human population (Steiger and Heuss, 2020). In 2022 a documentary titled “The invisible extinction” premiered at CPH:DOX explain the project by its founders https://www.theinvisibleextinction.com/. Such samples and collections are to be made available for future resuscitation, culturing, and research based on clearly defined rules such as those established by a dedicated international treaty—a human microbial “Noah’s Ark.”

## Concluding statement

The human microbiome has clear implications for health and disease and is getting popularized in clinical and translational studies. Many limitations of microbiome studies include the fact that the large and complex data sets require specific training for effective analysis which is not usually available worldwide, corresponding to the need in representation of biological samples. Low- and middle-income countries -where most human microbial diversity and health problems reside-requires a significant investment in training in bioinformatics so that scientists at ease utilizing and creating projects involving sequence data. Open science, including meta-analysis of previously published microbiome data can also pave the way to new hypotheses and knowledge.

For this to happen it is essential that only high-quality metadata and raw sequence data are used and openly accessible. Transparency and reproducibility should also be enhanced and required from published papers, with the use of standard methodologies such as protocols suggested by international consortia such as The Earth Microbiome Project or the international Human Microbiome Standards and the use of microbiome data management tools (e.g., QIITA) (Gonzalez et al., 2018; Bokulich et al., 2020). Efforts to standardize procedures and analyses as well as to promote equity and inclusion in this Frontier microbial science are underway and many resources that can help in conducting microbiome, research have been very well summarized in (Foxx et al., 2021; Marcos-Zambrano et al., 2021). Certainly, microbiome efforts from around the world are showing unique characteristics of the microbiota dependent on lifestyles and geographical areas.

Microbiome research is transforming our understanding of human biology. There are still there are still many answers that remain unanswered, including detailed microbiome transmission in body sites, lifestyle impacts to microbiome transmission, how microbiomes evolve and stabilize after antibiotics, population resilience and even how other members such as Fungi, Archaea or even the virome respond to changes in the bacterial biota, from perturbations to new probiotic therapeutics and if and how these dynamic changes continue longitudinally through time. Although there is still much research to be done to understand the mechanistic links between the microbiome and disease, this branch of the microbial sciences is opening vast opportunities for therapeutic treatments and improving human health.

## Figures and Tables

**TABLE 1:** Summary of the main bacterial taxa at human body sites.

Body site	Main taxa in healthy individuals	Main alterations in disease	Associated diseases	References
Vagina	*Lactobacillus crispatus, L. iners, L. gasseri, L. jensenii Streptococcus, Bifidobacterium*	↑ *Sneathia, Atopobium, Gardnerella*	Bacterial vaginosis, Vulvovaginal infections (RVVI), HPV infections and cervical cancer	Felten et al. (1999), Zhou et al. (2007), Di Paola et al. (2017)
	Very low abundance of anaerobes, *Prevotella*, *Atopobium*, Sneathia Gardnerella	↓ Lactobacilli	Symptoms associated with these include discomfort, odor, discharge, infertility, and, if pregnant, could even lead to miscarriages	
Skin	*Staphylococcus*, *Propionibacterium*, *Corynebacterium,* and *Streptococcus*	↑ *S. aureus, S. epidermidis, P. acnes,* Proteobacteria	Psoriasis, atopic dermatitis, systemic lupus erythematosus and alopecia	Chang et al. (2018), Ho et al. (2019), Paller et al. (2019), Bay et al. (2020), Huang et al. (2020)
		↓ *Acinetobacter Cutibacterium, Propionibacterium*, *Corynebacterium,* and *Staphylococcus*		
Eye	*Staphylococcus*, *Propionibacterium*, and *Pseudomonas*	↑*Delftia* and *Bacteroides*	Keratoconjunctivitis, mucosa-associated lymphoid tissue (MALT) lymphoma, and high glucose levels on the ocular surface due to diabetes	Asao et al. (2019), Li et al. (2019), Suzuki et al. (2020)
		↓Proteobacteria and Acinetobacter		
Ear	*Corynebacterium*, *Staphylococcus*, and *Propionibacterium*	↑ *Haemophilus*, *Alloiococcus Staphylococcus, Turicella, Moraxella, Streptococcus* and *Stenotrophomonas*	Otitis media infections: Acute Otitis Media (AOM) or Chronic Otitis Media with Effusion (COME)	Lappan et al. (2018), Jervis-Bardy et al. (2019), Kolbe et al. (2019)
Nasopharyngeal tract	*Corynebacteriaceae*, *Staphylococcaceae*, *Peptoniphilaceae*, *Carnobacteriacea*, *Staphylococcus*, *Corynebacterium*, *Alloiococcus*, *Haemophilus*, *Streptococcus, Granulicatella,* and *Moraxella*	↑ *Streptococcus, Haemophilus, Moraxella,* Proteobacteria, *Escherichia, Roseateles,* and *Pseudomonas*	Asthma, influenza A virus (IAV), bronchiolitis, and rhinosinusitis acute respiratory illness (ARI)	Teo et al. (2015), Stewart et al. (2017), Copeland et al. (2018), Wen et al. (2018), Kang and Kang, (2021)
		↓*Corynebacterium, Moraxella* and *Dolosigranulum*		
Oral	*Streptococcus*, *Gemella*, *Abiotrophia*, *Granulicatella*, *Rothia*, *Neisseria,* and *Prevotella*	↑ *Porphyromonas, Tannerella, Prevotella, Filifactor*	Dental cavities, gingivitis, periodontitis, oral cancer	Dewhirst et al. (2010), Crielaard et al. (2011), Huang et al. (2011), Kennedy et al. (2019), Sulyanto et al. (2019)
Gastrointestinal tract	*Clostridium*, *Bacteroides*, *Lactobacillus*, *Coprobacillus*, *Escherichia/Shigella, Bi*fi*dobacterium*, *Faecalibacterium prausnitzii*, *Eubacterium rectale*, *Akkermansia muciniphila*, *Enterococcus*, *Streptococcus*, *Veillonella*, *Prevotella*, *Helicobacter pylori*, *Stenotrophomonas*, *Lactococcus, Bacillus*, *Solibacillus*, *Pseudomonas*, *Arthrobacter*, *Lysinibacillus*	↑ *Veillonella*, *Fusobacterium*, *Prevotella* and *Gemella*, *Parvimonas* and other Proteobacteria	Gastroesophageal reflux disease (GERD), Barrett’s esophagus, or esophageal carcinoma, appendicitis	Pei et al. (2004), Maldonado-Contreras et al. (2011), Zoetendal et al. (2012), Guinane et al. (2013), Liu et al. (2013), Khan et al. (2014), Angelakis et al. (2015), Sundin et al. (2017), Gong et al. (2019), Fan et al. (2020), James et al. (2020)
		↓ *Bacteroides, Eubacterium rectale*, *Faecalibacterium prausnitzii*, *Akkermansia muciniphila* and Spirochaetes		

## References

[R1] Aabed K, Bhat RS, Al-Dbass A, Moubayed N, Algahtani N, Merghani NM, (2019). Bee pollen and propolis improve neuroinflammation and dysbiosis induced by propionic acid, a short chain fatty acid in a rodent model of autism. Lipids Health Dis. 18, 200. doi:10.1186/s12944-019-1150-0 31733650 PMC6858724

[R2] Alderete TL, Chen Z, Toledo-Corral CM, Contreras ZA, Kim JS, Habre R, (2018). Ambient and traffic-related air pollution exposures as novel risk factors for metabolic dysfunction and type 2 diabetes. Curr. Epidemiol. Rep 5, 79–91. doi:10.1007/s40471-018-0140-5 30319933 PMC6178230

[R3] Anand S, Mandal S, and Tomar SK (2019). Effect of Lactobacillus rhamnosus NCDC 298 with FOS in combination on viability and toxin production of enterotoxigenic *Escherichia coli*. Probiotics Antimicrob. Proteins 11, 23–29. doi:10.1007/s12602-017-9327-1 28948579

[R4] Angelakis E, Armougom F, Carrière F, Bachar D, Laugier R, Lagier J-C, (2015). A metagenomic investigation of the duodenal microbiota reveals links with obesity. PLoS One 10, e0137784. doi:10.1371/journal.pone.0137784 26356733 PMC4565581

[R5] Arnold IC, Dehzad N, Reuter S, Martin H, Becher B, Taube C, (2011). *Helicobacter pylori* infection prevents allergic asthma in mouse models through the induction of regulatory T cells. J. Clin. Invest 121, 3088–3093. doi:10.1172/JCI45041 21737881 PMC3148731

[R6] Arweiler NB, and Netuschil L (2016). The oral microbiota. Adv. Exp. Med. Biol 902, 45–60. doi:10.1007/978-3-319-31248-4_4 27161350

[R7] Asao K, Hashida N, Ando S, Motooka D, Kurakami H, Nakamura S, (2019). Conjunctival dysbiosis in mucosa-associated lymphoid tissue lymphoma. Sci. Rep 9, 8424. doi:10.1038/s41598-019-44861-5 31182732 PMC6557838

[R8] Auriemma RS, Scairati R, del Vecchio G, Liccardi A, Verde N, Pirchio R, (2021). The vaginal microbiome: A long urogenital colonization throughout woman life. Front. Cell. Infect. Microbiol 11, 686167. doi:10.3389/fcimb.2021.686167 34295836 PMC8290858

[R9] Baedke J, Fábregas-Tejeda A, and Nieves Delgado A (2020). The holobiont concept before Margulis. J. Exp. Zool. B Mol. Dev. Evol 334, 149–155. doi:10.1002/jez.b.22931 32039567

[R10] Baquero F, and Nombela C (2012). The microbiome as a human organ. Clin. Microbiol. Infect 18, 2–4. doi:10.1111/j.1469-0691.2012.03916.x 22647038

[R11] Barrett E, Ross RP, O’Toole PW, Fitzgerald GF, and Stanton C (2012). γ-Aminobutyric acid production by culturable bacteria from the human intestine. J. Appl. Microbiol 113, 411–417. doi:10.1111/j.1365-2672.2012.05344.x 22612585

[R12] Bay L, Barnes CJ, Fritz BG, Thorsen J, Restrup MEM, Rasmussen L, (2020). Universal dermal microbiome in human skin. mBio 11, e02945–19. doi:10.1128/mBio.02945-19 32047129 PMC7018652

[R13] Benmarhnia T, Delpla I, Schwarz L, Rodriguez MJ, and Levallois P (2018). Heterogeneity in the relationship between disinfection by-products in drinking water and cancer: A systematic review. Int. J. Environ. Res. Public Health 15, 979. doi:10.3390/ijerph15050979 29757939 PMC5982018

[R14] Bever CS, Rand AA, Nording M, Taft D, Kalanetra KM, Mills DA, (2018). Effects of triclosan in breast milk on the infant fecal microbiome. Chemosphere 203, 467–473. doi:10.1016/j.chemosphere.2018.03.186 29635158 PMC5915298

[R15] Bhatt AP, Pellock SJ, Biernat KA, Walton WG, Wallace BD, Creekmore BC, (2020). Targeted inhibition of gut bacterial β-glucuronidase activity enhances anticancer drug efficacy. Proc. Natl. Acad. Sci.U. S. A 117, 7374–7381. doi:10.1073/pnas.1918095117 32170007 PMC7132129

[R16] Bhattacharya S, Manna P, Gachhui R, and Sil PC (2013). D-saccharic acid 1, 4-lactone protects diabetic rat kidney by ameliorating hyperglycemia-mediated oxidative stress and renal inflammatory cytokines via NF-κB and PKC signaling. Toxicol. Appl. Pharmacol 267, 16–29. doi:10.1016/j.taap.2012.12.005 23261973

[R17] Bian X, Tu P, Chi L, Gao B, Ru H, Lu K, (2017). Saccharin induced liver inflammation in mice by altering the gut microbiota and its metabolic functions. Food Chem. Toxicol 107, 530–539. doi:10.1016/j.fct.2017.04.045 28472674 PMC5647777

[R18] Black M, Bhattacharya S, Philip S, Norman JE, and McLernon DJ (2016). Planned repeat cesarean section at term and adverse childhood health outcomes: A record-linkage study. PLoS Med. 13, e1001973. doi:10.1371/journal.pmed.1001973 26978456 PMC4792387

[R19] Blaser MJ, Dominguez-Bello MG, Contreras M, Magris M, Hidalgo G, Estrada I, (2013). Distinct cutaneous bacterial assemblages in a sampling of South American Amerindians and US residents. ISME J. 7, 85–95. doi:10.1038/ismej.2012.81 22895161 PMC3526177

[R20] Blaser MJ, and Falkow S (2009). What are the consequences of the disappearing human microbiota? Nat. Rev. Microbiol 7, 887–894. doi:10.1038/nrmicro2245 19898491 PMC9354563

[R21] Boem F, and Amedei A (2019). Healthy axis: Towards an integrated view of the gut-brain health. World J. Gastroenterol 25, 3838–3841. doi:10.3748/wjg.v25.i29.3838 31413521 PMC6689813

[R22] Bokulich NA, Ziemski M, Robeson MS, and Kaehler BD (2020). Measuring the microbiome: Best practices for developing and benchmarking microbiomics methods. Comput. Struct. Biotechnol. J 18, 4048–4062. doi:10.1016/j.csbj.2020.11.049 33363701 PMC7744638

[R23] Bordenstein SR, and Theis KR (2015). Host biology in light of the microbiome: Ten principles of holobionts and hologenomes. PLoS Biol. 13, e1002226. doi:10.1371/journal.pbio.1002226 26284777 PMC4540581

[R24] Bouslimani A, da Silva R, Kosciolek T, Janssen S, Callewaert C, Amir A, (2019). The impact of skin care products on skin chemistry and microbiome dynamics. BMC Biol. 17, 47. doi:10.1186/s12915-019-0660-6 31189482 PMC6560912

[R25] Brandt K (1881). Über das Zusammenleben von Algen und Tieren. Biol. Centallblatt 1, 524–527.

[R26] Burrello C, Giuffrè MR, Macandog AD, Diaz-Basabe A, Cribiù FM, Lopez G, (2019). Fecal microbiota transplantation controls murine chronic intestinal inflammation by modulating immune cell functions and gut microbiota composition. Cells 8, E517. doi:10.3390/cells8060517 PMC6628315 31142049

[R27] Cammarota G, Ianiro G, Tilg H, Rajilić-Stojanović M, Kump P, Satokari R, (2017). European consensus conference on faecal microbiota transplantation in clinical practice. Gut 66, 569–580. doi:10.1136/gutjnl-2016-313017 28087657 PMC5529972

[R28] Cano RJ, Rivera-Perez J, Toranzos GA, Santiago-Rodriguez TM, Narganes-Storde YM, Chanlatte-Baik L, (2014). Paleomicrobiology: Revealing fecal microbiomes of ancient indigenous cultures. PLoS One 9, e106833. doi:10.1371/journal.pone.0106833 25207979 PMC4160228

[R29] Capuco A, Urits I, Hasoon J, Chun R, Gerald B, Wang JK, (2020). Current perspectives on gut microbiome dysbiosis and depression. Adv. Ther 37, 1328–1346. doi:10.1007/s12325-020-01272-7 32130662 PMC7140737

[R30] Chang H-W, Kim K-H, Nam Y-D, Roh SW, Kim M-S, Jeon CO, (2008). Analysis of yeast and archaeal population dynamics in kimchi using denaturing gradient gel electrophoresis. Int. J. Food Microbiol 126, 159–166. doi:10.1016/j.ijfoodmicro.2008.05.013 18562030

[R31] Chang H-W, Yan D, Singh R, Liu J, Lu X, Ucmak D, (2018). Alteration of the cutaneous microbiome in psoriasis and potential role in Th17 polarization. Microbiome 6, 154. doi:10.1186/s40168-018-0533-1 30185226 PMC6125946

[R32] Chassaing B, Van de Wiele T, De Bodt J, Marzorati M, and Gewirtz AT (2017). Dietary emulsifiers directly alter human microbiota composition and gene expression *ex vivo* potentiating intestinal inflammation. Gut 66, 1414–1427. doi:10.1136/gutjnl-2016-313099 28325746 PMC5940336

[R33] Chaudhari DS, Dhotre DP, Agarwal DM, Gaike AH, Bhalerao D, Jadhav P, (2020). Gut, oral and skin microbiome of Indian patrilineal families reveal perceptible association with age. Sci. Rep 10, 5685. doi:10.1038/s41598-020-62195-5 32231240 PMC7105498

[R34] Chen C, Hemme C, Beleno J, Shi ZJ, Ning D, Qin Y, (2018). Oral microbiota of periodontal health and disease and their changes after nonsurgical periodontal therapy. ISME J. 12, 1210–1224. doi:10.1038/s41396-017-0037-1 29339824 PMC5932080

[R35] Chen X, Sun H, Jiang F, Shen Y, Li X, Hu X, (2020). Alteration of the gut microbiota associated with childhood obesity by 16S rRNA gene sequencing. PeerJ 8, e8317. doi:10.7717/peerj.8317 31976177 PMC6968493

[R36] Chien AL, Tsai J, Leung S, Mongodin EF, Nelson AM, Kang S, (2019). Association of systemic antibiotic treatment of acne with skin microbiota characteristics. JAMA Dermatol. 155, 425–434. doi:10.1001/jamadermatol.2018.5221 30758497 PMC6459106

[R37] Chiu L, and Gilbert SF (2015). The birth of the holobiont: Multi-species birthing through mutual scaffolding and niche construction. Biosemiotics 8, 191–210. doi:10.1007/s12304-015-9232-5

[R38] Clemente JC, Pehrsson EC, Blaser MJ, Sandhu K, Gao Z, Wang B, (2015). The microbiome of uncontacted Amerindians. Sci. Adv 1, e1500183. doi:10.1126/sciadv.1500183 26229982 PMC4517851

[R39] Combellick JL, Shin H, Shin D, Cai Y, Hagan H, Lacher C, (2018). Differences in the fecal microbiota of neonates born at home or in the hospital. Sci. Rep 8, 15660. doi:10.1038/s41598-018-33995-7 30353125 PMC6199260

[R40] Copeland E, Leonard K, Carney R, Kong J, Forer M, Naidoo Y, (2018). Chronic rhinosinusitis: Potential role of microbial dysbiosis and recommendations for sampling sites. Front. Cell. Infect. Microbiol 8, 57. doi:10.3389/fcimb.2018.00057 29541629 PMC5836553

[R41] Coton M, Pawtowski A, Taminiau B, Burgaud G, Deniel F, Coulloumme-Labarthe L, (2017). Unraveling microbial ecology of industrial-scale Kombucha fermentations by metabarcoding and culture-based methods. FEMS Microbiol. Ecol 93, fix048. doi:10.1093/femsec/fix048 28430940

[R42] Crielaard W, Zaura E, Schuller AA, Huse SM, Montijn RC, Keijser BJ, (2011). Exploring the oral microbiota of children at various developmental stages of their dentition in the relation to their oral health. BMC Med. Genomics 4, 22. doi:10.1186/1755-8794-4-22 21371338 PMC3058002

[R43] De Filippo C, Di Paola M, Ramazzotti M, Albanese D, Pieraccini G, Banci E, (2017). Diet, environments, and gut microbiota. A preliminary investigation in children living in rural and urban Burkina Faso and Italy. Front. Microbiol 8, 1979. doi:10.3389/fmicb.2017.01979 29081768 PMC5645538

[R44] Delport S (2019). Global epidemiology of use of and disparities in caesarean sections. Lancet 394, 23–24. doi:10.1016/S0140-6736(19)30717-2 31282354

[R45] Derilus D, Godoy-Vitorino F, Rosado H, Agosto E, Dominguez-Bello MG, Cavallin H, (2020). An in-depth survey of the microbial landscape of the walls of a neonatal operating room. PLoS One 15, e0230957. doi:10.1371/journal.pone.0230957 32243474 PMC7122808

[R46] Dewhirst FE, Chen T, Izard J, Paster BJ, Tanner ACR, Yu W-H, (2010). The human oral microbiome. J. Bacteriol 192, 5002–5017. doi:10.1128/JB.00542-10 20656903 PMC2944498

[R47] Di Paola M, Sani C, Clemente AM, Iossa A, Perissi E, Castronovo G, (2017). Characterization of cervico-vaginal microbiota in women developing persistent high-risk Human Papillomavirus infection. Sci. Rep 7, 10200. doi:10.1038/s41598-017-09842-6 28860468 PMC5579045

[R48] Dominguez-Bello MG, Costello EK, Contreras M, Magris M, Hidalgo G, Fierer N, (2010). Delivery mode shapes the acquisition and structure of the initial microbiota across multiple body habitats in newborns. Proc. Natl. Acad. Sci. U. S. A 107, 11971–11975. doi:10.1073/pnas.1002601107 20566857 PMC2900693

[R49] Dominguez-Bello MG, De Jesus-Laboy KM, Shen N, Cox LM, Amir A, Gonzalez A, (2016). Partial restoration of the microbiota of cesarean-born infants via vaginal microbial transfer. Nat. Med 22, 250–253. doi:10.1038/nm.4039 26828196 PMC5062956

[R50] Dominguez-Bello MG, and Godoy-Vitorino F (2013). “Infant microbiome,” in Encyclopedia of metagenomics. Editor Nelson K (New York, NY): Springer), 280–285.

[R51] Dominguez-Bello MG, Godoy-Vitorino F, Knight R, and Blaser MJ (2019). Role of the microbiome in human development. Gut 68, 1108–1114. doi:10.1136/gutjnl-2018-317503 30670574 PMC6580755

[R52] Dumitrescu L, Popescu-Olaru I, Cozma L, Tulbă D, Hinescu ME, Ceafalan LC, (2018). Oxidative stress and the microbiota-gut-brain Axis. Oxid. Med. Cell. Longev 2018, 2406594. doi:10.1155/2018/2406594 PMC6304899 30622664

[R53] Eckburg PB, Bik EM, Bernstein CN, Purdom E, Dethlefsen L, Sargent M, (2005). Diversity of the human intestinal microbial flora. Science 308, 1635–1638. doi:10.1126/science.1110591 15831718 PMC1395357

[R54] El-Salhy M, Kristoffersen AB, Valeur J, Casen C, Hatlebakk JG, Gilja OH, (2022). Long-term effects of fecal microbiota transplantation (FMT) in patients with irritable bowel syndrome. Neurogastroenterol. Motil 34, e14200. doi:10.1111/nmo.14200 34145677

[R55] Erny D, Dokalis N, Mezö C, Castoldi A, Mossad O, Staszewski O, (2021). Microbiota-derived acetate enables the metabolic fitness of the brain innate immune system during health and disease. Cell Metab. 33, 2260–2276.e7. doi:10.1016/j.cmet.2021.10.010 34731656

[R56] Estadella D, da Penha Oller do Nascimento CM, Oyama LM, Ribeiro EB, Dâmaso AR, de Piano A, (2013). Lipotoxicity: Effects of dietary saturated and transfatty acids. Mediat. Inflamm 2013, 137579. doi:10.1155/2013/137579 PMC3572653 23509418

[R57] Fan H-N, Zhu P, Lu Y-M, Guo J-H, Zhang J, Qu G-Q, (2020). Mild changes in the mucosal microbiome during terminal ileum inflammation. Microb. Pathog 142, 104104. doi:10.1016/j.micpath.2020.104104 32120004

[R58] Fava F, Gitau R, Griffin BA, Gibson GR, Tuohy KM, Lovegrove JA, (2013). The type and quantity of dietary fat and carbohydrate alter faecal microbiome and short-chain fatty acid excretion in a metabolic syndrome “at-risk” population. Int. J. Obes 37, 216–223. doi:10.1038/ijo.2012.33 22410962

[R59] Felten A, Barreau C, Bizet C, Lagrange PH, and Philippon A (1999). Lactobacillus species identification, H2O2 production, and antibiotic resistance and correlation with human clinical status. J. Clin. Microbiol 37, 729–733. doi:10.1128/JCM.37.3.729-733.1999 9986841 PMC84537

[R60] Foster K, Schluter J, Coyte K, and Rakoff-Nahoum S (2017). The evolution of the host microbiome as an ecosystem on a leash. Nature 548, 43–51. doi:10.1038/nature23292 28770836 PMC5749636

[R61] Fouladi F, Bailey MJ, Patterson WB, Sioda M, Blakley IC, Fodor AA, (2020). Air pollution exposure is associated with the gut microbiome as revealed by shotgun metagenomic sequencing. Environ. Int 138, 105604. doi:10.1016/j.envint.2020.105604 32135388 PMC7181344

[R62] Foxx AJ, Franco Meléndez KP, Hariharan J, Kozik AJ, Wattenburger CJ, Godoy-Vitorino F, (2021). Advancing equity and inclusion in microbiome research and training. mSystems 6, e0115121. doi:10.1128/mSystems.01151-21 34636663 PMC8510545

[R63] Funkhouser LJ, and Bordenstein SR (2013). Mom knows best: The universality of maternal microbial transmission. PLoS Biol. 11, e1001631. doi:10.1371/journal.pbio.1001631 23976878 PMC3747981

[R64] Gaggìa F, Baffoni L, Galiano M, Nielsen DS, Jakobsen RR, Castro-Mejía JL, (2018). Kombucha beverage from green, black and rooibos teas: A comparative study looking at microbiology, chemistry and antioxidant activity. Nutrients 11, E1. doi:10.3390/nu11010001 PMC6356548 30577416

[R65] Gaike AH, Paul D, Bhute S, Dhotre DP, Pande P, Upadhyaya S, (2020). The gut microbial diversity of newly diagnosed diabetics but not of prediabetics is significantly different from that of healthy nondiabetics. mSystems 5, e00578–19. doi:10.1128/mSystems.00578-19 32234773 PMC7112960

[R66] Garcia-Gutierrez E, Narbad A, and Rodríguez JM (2020). Autism spectrum disorder associated with gut microbiota at immune, metabolomic, and neuroactive level. Front. Neurosci 14, 578666. doi:10.3389/fnins.2020.578666 33117122 PMC7578228

[R67] Gilbert SF, Sapp J, and Tauber AI (2012). A symbiotic view of life: We have never been individuals. Q. Rev. Biol 87, 325–341. doi:10.1086/668166 23397797

[R68] Godoy-Vitorino F (2019). Human microbial ecology and the rising new medicine. Ann. Transl. Med 7, 342. doi:10.21037/atm.2019.06.56 31475212 PMC6694241

[R69] Godoy-Vitorino F, Romaguera J, Zhao C, Vargas-Robles D, Ortiz-Morales G, Vázquez-Sánchez F, (2018). Cervicovaginal fungi and bacteria associated with cervical intraepithelial neoplasia and high-risk human papillomavirus infections in a hispanic population. Front. Microbiol 9, 2533. doi:10.3389/fmicb.2018.02533 30405584 PMC6208322

[R70] Gomez A, Petrzelkova KJ, Burns MB, Yeoman CJ, Amato KR, Vlckova K, (2016). Gut microbiome of coexisting BaAka pygmies and Bantu reflects gradients of traditional subsistence patterns. Cell Rep. 14, 2142–2153. doi:10.1016/j.celrep.2016.02.013 26923597

[R71] Gong J, Li L, Zuo X, and Li Y (2019). Change of the duodenal mucosa-associated microbiota is related to intestinal metaplasia. BMC Microbiol. 19, 275. doi:10.1186/s12866-019-1666-5 31815623 PMC6900849

[R72] Gonzalez A, Navas-Molina JA, Kosciolek T, McDonald D, Vázquez-Baeza Y, Ackermann G, (2018). Qiita: Rapid, web-enabled microbiome meta-analysis. Nat. Methods 15, 796–798. doi:10.1038/s41592-018-0141-9 30275573 PMC6235622

[R73] Gopalakrishnan V, Spencer CN, Nezi L, Reuben A, Andrews MC, Karpinets TV, (2018). Gut microbiome modulates response to anti-PD-1 immunotherapy in melanoma patients. Science 359, 97–103. doi:10.1126/science.aan4236 29097493 PMC5827966

[R74] Gregory KE, Samuel BS, Houghteling P, Shan G, Ausubel FM, Sadreyev RI, (2016). Influence of maternal breast milk ingestion on acquisition of the intestinal microbiome in preterm infants. Microbiome 4, 68. doi:10.1186/s40168-016-0214-x 28034306 PMC5200970

[R75] Guerrero-Preston R, Godoy-Vitorino F, Jedlicka A, Rodríguez-Hilario A, González H, Bondy J, (2016). 16S rRNA amplicon sequencing identifies microbiota associated with oral cancer, human papilloma virus infection and surgical treatment. Oncotarget 7, 51320–51334. doi:10.18632/oncotarget.9710 27259999 PMC5239478

[R76] Guinane CM, Tadrous A, Fouhy F, Ryan CA, Dempsey EM, Murphy B, (2013). Microbial composition of human appendices from patients following appendectomy. mBio 4, e00366–12. doi:10.1128/mBio.00366-12 PMC3551545 23322636

[R77] Guo Y, Xie J-P, Deng K, Li X, Yuan Y, Xuan Q, (2019). Prophylactic effects of Bifidobacterium adolescentis on anxiety and depression-like phenotypes after chronic stress: A role of the gut microbiota-inflammation Axis. Front. Behav. Neurosci 13, 126. doi:10.3389/fnbeh.2019.00126 31275120 PMC6591489

[R78] Gupta AK, and Kohli Y (2004). Prevalence of Malassezia species on various body sites in clinically healthy subjects representing different age groups. Med. Mycol 42, 35–42. doi:10.1080/13693780310001610056 14982112

[R79] Haiser HJ, Gootenberg DB, Chatman K, Sirasani G, Balskus EP, Turnbaugh PJ, (2013). Predicting and manipulating cardiac drug inactivation by the human gut bacterium Eggerthella lenta. Science 341, 295–298. doi:10.1126/science.1235872 23869020 PMC3736355

[R80] Haran JP, Bhattarai SK, Foley SE, Dutta P, Ward DV, Bucci V, (2019). Alzheimer’s disease microbiome is associated with dysregulation of the anti-inflammatory P-glycoprotein pathway. mBio 10, e00632–19. doi:10.1128/mBio.00632-19 31064831 PMC6509190

[R81] Hegde S, and Munshi AK (1998). Influence of the maternal vaginal microbiota on the oral microbiota of the newborn. J. Clin. Pediatr. Dent 22, 317–321. 9796502

[R82] Helve O, Dikareva E, Stefanovic V, Kolho K-L, Salonen A, de Vos WM, (2021). Protocol for oral transplantation of maternal fecal microbiota to newborn infants born by cesarean section. Star. Protoc 2, 100271. doi:10.1016/j.xpro.2020.100271 33511356 PMC7817495

[R83] Henning SM, Yang J, Shao P, Lee R-P, Huang J, Ly A, (2017). Health benefit of vegetable/fruit juice-based diet: Role of microbiome. Sci. Rep 7, 2167. doi:10.1038/s41598-017-02200-6 28526852 PMC5438379

[R84] Ho BS-Y, Ho EXP, Chu CW, Ramasamy S, Bigliardi-Qi M, de Sessions PF, (2019). Microbiome in the hair follicle of androgenetic alopecia patients. PLoS One 14, e0216330. doi:10.1371/journal.pone.0216330 31050675 PMC6499469

[R85] Hoffmann C, Dollive S, Grunberg S, Chen J, Li H, Wu GD, (2013). Archaea and fungi of the human gut microbiome: Correlations with diet and bacterial residents. PLoS One 8, e66019. doi:10.1371/journal.pone.0066019 23799070 PMC3684604

[R86] Holzer P, and Farzi A (2014). Neuropeptides and the microbiota-gut-brain axis. Adv. Exp. Med. Biol 817, 195–219. doi:10.1007/978-1-4939-0897-4_9 24997035 PMC4359909

[R87] Huang C, Yi X, Long H, Zhang G, Wu H, Zhao M, (2020). Disordered cutaneous microbiota in systemic lupus erythematosus. J. Autoimmun 108, 102391. doi:10.1016/j.jaut.2019.102391 31883828

[R88] Huang S, Yang F, Zeng X, Chen J, Li R, Wen T, (2011). Preliminary characterization of the oral microbiota of Chinese adults with and without gingivitis. BMC Oral Health 11, 33. doi:10.1186/1472-6831-11-33 22152152 PMC3254127

[R89] Huang Y, Shi X, Li Z, Shen Y, Shi X, Wang L, (2018). Possible association of Firmicutes in the gut microbiota of patients with major depressive disorder. Neuropsychiatr. Dis. Treat 14, 3329–3337. doi:10.2147/NDT.S188340 30584306 PMC6284853

[R90] Iglesias-Vázquez L, Van Ginkel Riba G, Arija V, and Canals J (2020). Composition of gut microbiota in children with autism spectrum disorder: A systematic review and meta-analysis. Nutrients 12, E792. doi:10.3390/nu12030792 PMC7146354 32192218

[R91] Indiani CMDSP, Rizzardi KF, Castelo PM, Ferraz LFC, Darrieux M, Parisotto TM, (2018). Childhood obesity and firmicutes/bacteroidetes ratio in the gut microbiota: A systematic review. Child. Obes 14, 501–509. doi:10.1089/chi.2018.0040 30183336

[R92] James KR, Gomes T, Elmentaite R, Kumar N, Gulliver EL, King HW, (2020). Distinct microbial and immune niches of the human colon. Nat. Immunol 21, 343–353. doi:10.1038/s41590-020-0602-z 32066951 PMC7212050

[R93] Jeong SH, Jung JY, Lee SH, Jin HM, and Jeon CO (2013). Microbial succession and metabolite changes during fermentation of dongchimi, traditional Korean watery kimchi. Int. J. Food Microbiol 164, 46–53. doi:10.1016/j.ijfoodmicro.2013.03.016 23587713

[R94] Jervis-Bardy J, Leong LEX, Papanicolas LE, Ivey KL, Chawla S, Woods CM, (2019). Examining the evidence for an adult healthy middle ear microbiome. mSphere 4, e00456–19. doi:10.1128/mSphere.00456-19 31484741 PMC6731531

[R95] Jha AR, Davenport ER, Gautam Y, Bhandari D, Tandukar S, Ng KM, (2018). Gut microbiome transition across a lifestyle gradient in Himalaya. PLoS Biol. 16, e2005396. doi:10.1371/journal.pbio.2005396 30439937 PMC6237292

[R96] Jiao N, Baker SS, Nugent CA, Tsompana M, Cai L, Wang Y, (2018). Gut microbiome may contribute to insulin resistance and systemic inflammation in obese rodents: A meta-analysis. Physiol. Genomics 50, 244–254. doi:10.1152/physiolgenomics.00114.2017 29373083

[R97] Jo J-H, Kennedy EA, and Kong HH (2017). Topographical and physiological differences of the skin mycobiome in health and disease. Virulence 8, 324–333. doi:10.1080/21505594.2016.1249093 27754756 PMC5411233

[R98] Jung Y, Kim I, Mannaa M, Kim J, Wang S, Park I, (2019). Effect of Kombucha on gut-microbiota in mouse having non-alcoholic fatty liver disease. Food Sci. Biotechnol 28, 261–267. doi:10.1007/s10068-018-0433-y 30815318 PMC6365329

[R99] Kang HM, and Kang JH (2021). Effects of nasopharyngeal microbiota in respiratory infections and allergies. Clin. Exp. Pediatr 64, 543–551. doi:10.3345/cep.2020.01452 33872488 PMC8566799

[R100] Karen C, Shyu DJH, and Rajan KE (2021). Lactobacillus paracasei supplementation prevents early life stress-induced anxiety and depressive-like behavior in maternal separation model-possible involvement of microbiota-gut-brain Axis in differential regulation of MicroRNA124a/132 and glutamate receptors. Front. Neurosci 15, 719933. doi:10.3389/fnins.2021.719933 34531716 PMC8438336

[R101] Kaur H, Merchant M, Haque MM, and Mande SS (2020). Crosstalk between female gonadal hormones and vaginal microbiota across various phases of women’s gynecological lifecycle. Front. Microbiol 11, 551. doi:10.3389/fmicb.2020.00551 32296412 PMC7136476

[R102] Kennedy B, Peura S, Hammar U, Vicenzi S, Hedman A, Almqvist C, (2019). Oral microbiota development in early childhood. Sci. Rep 9, 19025. doi:10.1038/s41598-019-54702-0 31836727 PMC6911045

[R103] Khan MT, van Dijl JM, and Harmsen HJM (2014). Antioxidants keep the potentially probiotic but highly oxygen-sensitive human gut bacterium Faecalibacterium prausnitzii alive at ambient air. PLoS One 9, e96097. doi:10.1371/journal.pone.0096097 24798051 PMC4010535

[R104] Kim D-H, Kim H, Jeong D, Kang I-B, Chon J-W, Kim H-S, (2017). Kefir alleviates obesity and hepatic steatosis in high-fat diet-fed mice by modulation of gut microbiota and mycobiota: Targeted and untargeted community analysis with correlation of biomarkers. J. Nutr. Biochem 44, 35–43. doi:10.1016/j.jnutbio.2017.02.014 28384519

[R105] Kim H, Sitarik AR, Woodcroft K, Johnson CC, and Zoratti E (2019a). Birth mode, breastfeeding, pet exposure, and antibiotic use: Associations with the gut microbiome and sensitization in children. Curr. Allergy Asthma Rep 19, 22. doi:10.1007/s11882-019-0851-9 30859338 PMC7376540

[R106] Kim J-H, Kim K, and Kim W (2019b). Cream cheese-derived Lactococcus chungangensis CAU 28 modulates the gut microbiota and alleviates atopic dermatitis in BALB/c mice. Sci. Rep 9, 446. doi:10.1038/s41598-018-36864-5 30679532 PMC6345912

[R107] Kisuse J, La-Ongkham O, Nakphaichit M, Therdtatha P, Momoda R, Tanaka M, (2018). Urban diets linked to gut microbiome and metabolome alterations in children: A comparative cross-sectional study in Thailand. Front. Microbiol 9, 1345. doi:10.3389/fmicb.2018.01345 29988433 PMC6024022

[R108] Knudsen JK, Michaelsen TY, Bundgaard-Nielsen C, Nielsen RE, Hjerrild S, Leutscher P, (2021). Faecal microbiota transplantation from patients with depression or healthy individuals into rats modulates mood-related behaviour. Sci. Rep 11, 21869. doi:10.1038/s41598-021-01248-9 34750433 PMC8575883

[R109] Kolbe AR, Castro-Nallar E, Preciado D, and Pérez-Losada M (2019). Altered middle ear microbiome in children with chronic otitis media with effusion and respiratory illnesses. Front. Cell. Infect. Microbiol 9, 339. doi:10.3389/fcimb.2019.00339 31637220 PMC6787523

[R110] Margulis L and Fester R (Editors) (1991). Symbiosis as a source of evolutionary innovation: Speciation and morphogenesis (Cambridge, MA, USA: MIT Press). 11538111

[R111] Lacourt-Ventura MY, Vilanova-Cuevas B, Rivera-Rodríguez D, Rosario-Acevedo R, Miranda C, Maldonado-Martínez G, (2021). Soy and frequent dairy consumption with subsequent equol production reveals decreased gut health in a cohort of healthy Puerto Rican women. Int. J. Environ. Res. Public Health 18, 8254. doi:10.3390/ijerph18168254 34444002 PMC8391519

[R112] Langdon A, Crook N, and Dantas G (2016). The effects of antibiotics on the microbiome throughout development and alternative approaches for therapeutic modulation. Genome Med. 8, 39. doi:10.1186/s13073-016-0294-z 27074706 PMC4831151

[R113] Lappan R, Imbrogno K, Sikazwe C, Anderson D, Mok D, Coates H, (2018). A microbiome case-control study of recurrent acute otitis media identified potentially protective bacterial genera. BMC Microbiol. 18, 13. doi:10.1186/s12866-018-1154-3 29458340 PMC5819196

[R114] Le Roy CI, Kurilshikov A, Leeming ER, Visconti A, Bowyer RCE, Menni C, (2022). Yoghurt consumption is associated with changes in the composition of the human gut microbiome and metabolome. BMC Microbiol. 22, 39. doi:10.1186/s12866-021-02364-2 35114943 PMC8812230

[R115] Lebwohl B, Blaser MJ, Ludvigsson JF, Green PHR, Rundle A, Sonnenberg A, (2013). Decreased risk of celiac disease in patients with *Helicobacter pylori* colonization. Am. J. Epidemiol 178, 1721–1730. doi:10.1093/aje/kwt234 24124196 PMC3858109

[R116] Lecerf J-M, and Cani PD (2022). Nutrition et microbiote dans le diabète de type 2. De la symbiose à la dysfonction métabolique. Médecine Des. Mal. Métaboliques 16, 114–120. doi:10.1016/j.mmm.2022.01.002

[R117] Li SS, Zhu A, Benes V, Costea PI, Hercog R, Hildebrand F, (2016). Durable coexistence of donor and recipient strains after fecal microbiota transplantation. Science 352, 586–589. doi:10.1126/science.aad8852 27126044

[R118] Li S, Yi G, Peng H, Li Z, Chen S, Zhong H, (2019). How ocular surface microbiota debuts in type 2 diabetes mellitus. Front. Cell. Infect. Microbiol 9, 202. doi:10.3389/fcimb.2019.00202 31263683 PMC6590198

[R119] Liu N, Ando T, Ishiguro K, Maeda O, Watanabe O, Funasaka K, (2013). Characterization of bacterial biota in the distal esophagus of Japanese patients with reflux esophagitis and Barrett’s esophagus. BMC Infect. Dis 13, 130. doi:10.1186/1471-2334-13-130 23496929 PMC3599685

[R120] Lleal M, Sarrabayrouse G, Willamil J, Santiago A, Pozuelo M, Manichanh C, (2019). A single faecal microbiota transplantation modulates the microbiome and improves clinical manifestations in a rat model of colitis. EBioMedicine 48, 630–641. doi:10.1016/j.ebiom.2019.10.002 31628021 PMC6838378

[R121] Lokmer A, Aflalo S, Amougou N, Lafosse S, Froment A, Tabe FE, (2020). Response of the human gut and saliva microbiome to urbanization in Cameroon. Sci. Rep 10, 2856. doi:10.1038/s41598-020-59849-9 32071424 PMC7028744

[R122] Mahley RW, Weisgraber KH, and Huang Y (2009). Apolipoprotein E: Structure determines function, from atherosclerosis to alzheimer’s disease to AIDS. J. Lipid Res 50, S183–S188. doi:10.1194/jlr.R800069-JLR200 19106071 PMC2674716

[R123] Maldonado-Contreras A, Goldfarb KC, Godoy-Vitorino F, Karaoz U, Contreras M, Blaser MJ, (2011). Structure of the human gastric bacterial community in relation to *Helicobacter pylori* status. ISME J. 5, 574–579. doi:10.1038/ismej.2010.149 20927139 PMC3105737

[R124] Mandy M, and Nyirenda M (2018). Developmental origins of health and disease: The relevance to developing nations. Int. Health 10, 66–70. doi:10.1093/inthealth/ihy006 29528398 PMC5856182

[R125] Marcos-Zambrano LJ, Karaduzovic-Hadziabdic K, Loncar Turukalo T, Przymus P, Trajkovik V, Aasmets O, (2021). Applications of machine learning in human microbiome studies: A review on feature selection, biomarker identification, disease prediction and treatment. Front. Microbiol 12, 634511. doi:10.3389/fmicb.2021.634511 33737920 PMC7962872

[R126] Margulis L (1990). Words as battle cries: Symbiogenesis and the new field of endocytobiology. BioScience 40, 673. doi:10.2307/1311435 11541293

[R127] Mark Welch JL, Rossetti BJ, Rieken CW, Dewhirst FE, and Borisy GG (2016). Biogeography of a human oral microbiome at the micron scale. Proc. Natl. Acad. Sci. U. S. A 113, E791–E800. doi:10.1073/pnas.1522149113 26811460 PMC4760785

[R128] McDonald D, Hyde E, Debelius JW, Morton JT, Gonzalez A, Ackermann G, (2018). American gut: An open platform for citizen science microbiome research. mSystems 3, e00031–18. doi:10.1128/mSystems.00031-18 29795809 PMC5954204

[R129] McFall-Ngai M, Hadfield MG, Bosch TC, Carey HV, Domazet-Lošo T, Douglas AE, (2013). Animals in a bacterial world, a new imperative for the life sciences. Proc. Natl. Acad. Sci. U. S. A 110. 3229PMC3587249–36. Epub 2013 Feb 7. doi:10.1073/pnas.12185251109 PMC3587249 23391737

[R130] McGaughey KD, Yilmaz-Swenson T, Elsayed NM, Cruz DA, Rodriguiz RM, Kritzer MD, (2019). Relative abundance of Akkermansia spp. and other bacterial phylotypes correlates with anxiety- and depressive-like behavior following social defeat in mice. Sci. Rep 9, 3281. doi:10.1038/s41598-019-40140-5 30824791 PMC6397238

[R131] Mori N, Kano M, Masuoka N, Konno T, Suzuki Y, Miyazaki K, (2016). Effect of probiotic and prebiotic fermented milk on skin and intestinal conditions in healthy young female students. Biosci. Microbiota Food Health 35, 105–112. doi:10.12938/bmfh.2015-022 27508111 PMC4965514

[R132] Mueller NT, Bakacs E, Combellick J, Grigoryan Z, and Dominguez-Bello MG (2015). The infant microbiome development: Mom matters. Trends Mol. Med 21, 109–117. doi:10.1016/j.molmed.2014.12.002 25578246 PMC4464665

[R133] Nardelli C, Granata I, D’Argenio V, Tramontano S, Compare D, Guarracino MR, (2020). Characterization of the duodenal mucosal microbiome in obese adult subjects by 16S rRNA sequencing. Microorganisms 8, E485. doi:10.3390/microorganisms8040485 PMC7232320 32235377

[R134] Niccolai E, Boem F, Russo E, and Amedei A (2019). The Gut^−^Brain Axis in the neuropsychological disease model of obesity: A classical movie revised by the emerging director “microbiome. Nutrients 11, E156. doi:10.3390/nu11010156 PMC6356219 30642052

[R135] Nimgampalle M, and Kuna Y (2017). Anti-alzheimer properties of probiotic, Lactobacillus plantarum MTCC 1325 in alzheimer’s disease induced albino rats. J. Clin. Diagn. Res 11, KC01–KC05. doi:10.7860/JCDR/2017/26106.10428 PMC5620801 28969160

[R136] Obata Y, and Pachnis V (2016). The effect of microbiota and the immune system on the development and organization of the enteric nervous system. Gastroenterology 151, 836–844. doi:10.1053/j.gastro.2016.07.044 27521479 PMC5102499

[R137] Obregon-Tito AJ, Tito RY, Metcalf J, Sankaranarayanan K, Clemente JC, Ursell LK, (2015). Subsistence strategies in traditional societies distinguish gut microbiomes. Nat. Commun 6, 6505. doi:10.1038/ncomms7505 25807110 PMC4386023

[R138] Paller AS, Kong HH, Seed P, Naik S, Scharschmidt TC, Gallo RL, (2019). The microbiome in patients with atopic dermatitis. J. Allergy Clin. Immunol 143, 26–35. doi:10.1016/j.jaci.2018.11.015 30476499 PMC7163929

[R139] Panigrahi P, Parida S, Nanda NC, Satpathy R, Pradhan L, Chandel DS, (2017). A randomized synbiotic trial to prevent sepsis among infants in rural India. Nature 548, 407–412. doi:10.1038/nature23480 28813414

[R140] Park S, Ji Y, Jung H-Y, Park H, Kang J, Choi S-H, (2017). Lactobacillus plantarum HAC01 regulates gut microbiota and adipose tissue accumulation in a diet-induced obesity murine model. Appl. Microbiol. Biotechnol 101, 1605–1614. doi:10.1007/s00253-016-7953-2 27858139

[R141] Patra JK, Das G, Paramithiotis S, and Shin H-S (2016). Kimchi and other widely consumed traditional fermented foods of korea: A review. Front. Microbiol 7, 1493. doi:10.3389/fmicb.2016.01493 27733844 PMC5039233

[R142] Pei Z, Bini EJ, Yang L, Zhou M, Francois F, Blaser MJ, (2004). Bacterial biota in the human distal esophagus. Proc. Natl. Acad. Sci. U. S. A 101, 4250–4255. doi:10.1073/pnas.0306398101 15016918 PMC384727

[R143] Pigneur B, and Sokol H (2016). Fecal microbiota transplantation in inflammatory bowel disease: The quest for the holy grail. Mucosal Immunol. 9, 1360–1365. doi:10.1038/mi.2016.67 27461176

[R144] Pitocco D, Di Leo M, Tartaglione L, De Leva F, Petruzziello C, Saviano A, (2020). The role of gut microbiota in mediating obesity and diabetes mellitus. Eur. Rev. Med. Pharmacol. Sci 24, 1548–1562. doi:10.26355/eurrev_202002_20213 32096204

[R145] Pokusaeva K, Johnson C, Luk B, Uribe G, Fu Y, Oezguen N, (2017). GABA-producing Bifidobacterium dentium modulates visceral sensitivity in the intestine. Neurogastroenterol. Motil 29, e12904. doi:10.1111/nmo.12904 27458085 PMC5195897

[R146] Prado MR, Blandón LM, Vandenberghe LPS, Rodrigues C, Castro GR, Thomaz-Soccol V, (2015). Milk kefir: Composition, microbial cultures, biological activities, and related products. Front. Microbiol 6, 1177. doi:10.3389/fmicb.2015.01177 26579086 PMC4626640

[R147] Qu L, Ren J, Huang L, Pang B, Liu X, Liu X, (2018). Antidiabetic effects of Lactobacillus casei fermented yogurt through reshaping gut microbiota structure in type 2 diabetic rats. J. Agric. Food Chem 66, 12696–12705. doi:10.1021/acs.jafc.8b04874 30398060

[R148] Reyman M, van Houten MA, Watson RL, Chu MLJN, Arp K, de Waal WJ, (2022). Effects of early-life antibiotics on the developing infant gut microbiome and resistome: A randomized trial. Nat. Commun 13, 893. doi:10.1038/s41467-022-28525-z 35173154 PMC8850541

[R149] Rezac S, Kok CR, Heermann M, and Hutkins R (2018). Fermented foods as a dietary source of live organisms. Front. Microbiol 9, 1785. doi:10.3389/fmicb.2018.01785 30197628 PMC6117398

[R150] Ribado JV, Ley C, Haggerty TD, Tkachenko E, Bhatt AS, Parsonnet J, (2017). Household triclosan and triclocarban effects on the infant and maternal microbiome. EMBO Mol. Med 9, 1732–1741. doi:10.15252/emmm.201707882 29030459 PMC5709730

[R151] Ribière C, Peyret P, Parisot N, Darcha C, Déchelotte PJ, Barnich N, (2016). Oral exposure to environmental pollutant benzo[a]pyrene impacts the intestinal epithelium and induces gut microbial shifts in murine model. Sci. Rep 6, 31027. doi:10.1038/srep31027 27503127 PMC4977522

[R152] Rigo-Adrover MDM, van Limpt K, Knipping K, Garssen J, Knol J, Costabile A, (2018). Preventive effect of a synbiotic combination of galacto- and fructooligosaccharides mixture with Bifidobacterium breve M-16V in a model of multiple rotavirus infections. Front. Immunol 9, 1318. doi:10.3389/fimmu.2018.01318 29942312 PMC6004411

[R153] Rinninella E, Raoul P, Cintoni M, Franceschi F, Miggiano GAD, Gasbarrini A, (2019). What is the healthy gut microbiota composition? A changing ecosystem across age, environment, diet, and diseases. Microorganisms 7, E14. doi:10.3390/microorganisms7010014 PMC6351938 30634578

[R154] Roth W, Zadeh K, Vekariya R, Ge Y, and Mohamadzadeh M (2021). Tryptophan metabolism and gut-brain homeostasis. Int. J. Mol. Sci 22, 2973. doi:10.3390/ijms22062973 33804088 PMC8000752

[R155] Saad MJA, Santos A, and Prada PO (2016). Linking gut microbiota and inflammation to obesity and insulin resistance. Physiol. (Bethesda) 31, 283–293. doi:10.1152/physiol.00041.2015 27252163

[R156] Sakai Y, Seki N, Hamano K, Ochi H, Abe F, Masuda K, (2019). Prebiotic effect of two grams of lactulose in healthy Japanese women: A randomised, double-blind, placebo-controlled crossover trial. Benef. Microbes 10, 629–639. doi:10.3920/BM2018.0174 31131617

[R157] Sanjar F, Weaver AJ, Peacock TJ, Nguyen JQ, Brandenburg KS, Leung KP, (2020). Identification of metagenomics structure and function associated with temporal changes in rat (Rattus norvegicus) skin microbiome during health and cutaneous burn. J. Burn Care Res 41, 347–358. doi:10.1093/jbcr/irz165 31665423 PMC12711798

[R158] Santiago-Rodriguez TM, Fornaciari G, Luciani S, Dowd SE, Toranzos GA, Marota I, (2016). Taxonomic and predicted metabolic profiles of the human gut microbiome in pre-Columbian mummies. FEMS Microbiol. Ecol 92, fiw182. doi:10.1093/femsec/fiw182 27559027

[R159] Sarrabayrouse G, Landolfi S, Pozuelo M, Willamil J, Varela E, Clark A, (2020). Mucosal microbial load in Crohn’s disease: A potential predictor of response to faecal microbiota transplantation. EBioMedicine 51, 102611. doi:10.1016/j.ebiom.2019.102611 31901867 PMC6948165

[R160] Sasada T, Hinoi T, Saito Y, Adachi T, Takakura Y, Kawaguchi Y, (2015). Chlorinated water modulates the development of colorectal tumors with chromosomal instability and gut microbiota in apc-deficient mice. PLoS One 10, e0132435. doi:10.1371/journal.pone.0132435 26186212 PMC4505894

[R161] Schnorr SL, Candela M, Rampelli S, Centanni M, Consolandi C, Basaglia G, (2014). Gut microbiome of the Hadza hunter-gatherers. Nat. Commun 5, 3654. doi:10.1038/ncomms4654 24736369 PMC3996546

[R162] Schulfer A, and Blaser MJ (2015). Risks of antibiotic exposures early in life on the developing microbiome. PLoS Pathog. 11, e1004903. doi:10.1371/journal.ppat.1004903 26135581 PMC4489621

[R163] Scriven M, Dinan TG, Cryan JF, and Wall M (2018). Neuropsychiatric disorders: Influence of gut microbe to brain signalling. Diseases 6, E78. doi:10.3390/diseases6030078 PMC6163507 30200574

[R164] Seo YS, Lee H-B, Kim Y, and Park H-Y (2020). Dietary carbohydrate constituents related to gut dysbiosis and health. Microorganisms 8, 427. doi:10.3390/microorganisms8030427 32197401 PMC7143995

[R165] Serrano MG, Parikh HI, Brooks JP, Edwards DJ, Arodz TJ, Edupuganti L, (2019). Racioethnic diversity in the dynamics of the vaginal microbiome during pregnancy. Nat. Med 25, 1001–1011. doi:10.1038/s41591-019-0465-8 31142850 PMC6746180

[R166] Silva YP, Bernardi A, and Frozza RL (2020). The role of short-chain fatty acids from gut microbiota in gut-brain communication. Front. Endocrinol 11, 25. doi:10.3389/fendo.2020.00025 PMC7005631 32082260

[R167] Singh RK, Chang H-W, Yan D, Lee KM, Ucmak D, Wong K, (2017). Influence of diet on the gut microbiome and implications for human health. J. Transl. Med 15, 73. doi:10.1186/s12967-017-1175-y 28388917 PMC5385025

[R168] Slykerman RF, Hood F, Wickens K, Thompson JMD, Barthow C, Murphy R, (2017). Effect of Lactobacillus rhamnosus HN001 in pregnancy on postpartum symptoms of depression and anxiety: A randomised double-blind placebo-controlled trial. EBioMedicine 24, 159–165. doi:10.1016/j.ebiom.2017.09.013 28943228 PMC5652021

[R169] Sonnenburg ED, and Sonnenburg JL (2014). Starving our microbial self: The deleterious consequences of a diet deficient in microbiota-accessible carbohydrates. Cell Metab. 20, 779–786. doi:10.1016/j.cmet.2014.07.003 25156449 PMC4896489

[R170] Steiger D, and Heuss A (2020). Microbiota Vault - feasibility study, 43. Switzerland: Evalue Science AG.

[R171] Stewart CJ, Mansbach JM, Wong MC, Ajami NJ, Petrosino JF, Camargo CA, (2017). Associations of nasopharyngeal metabolome and microbiome with severity among infants with bronchiolitis. A multiomic analysis. Am. J. Respir. Crit. Care Med 196, 882–891. doi:10.1164/rccm.201701-0071OC 28530140 PMC5649976

[R172] Suárez J, and Stencel A (2020). A part-dependent account of biological individuality: Why holobionts are individuals and ecosystems simultaneously. Biol. Rev. Camb. Philos. Soc 95, 1308–1324. doi:10.1111/brv.12610 32406121

[R173] Suárez J (2018). The importance of symbiosis in philosophy of biology: An analysis of the current debate on biological individuality and its historical roots. Symbiosis 76, 77–96. doi:10.1007/s13199-018-0556-1

[R174] Sulyanto RM, Thompson ZA, Beall CJ, Leys EJ, and Griffen AL (2019). The predominant oral microbiota is acquired early in an organized pattern. Sci. Rep 9, 10550. doi:10.1038/s41598-019-46923-0 31332213 PMC6646312

[R175] Sundin OH, Mendoza-Ladd A, Zeng M, Diaz-Arévalo D, Morales E, Fagan BM, (2017). The human jejunum has an endogenous microbiota that differs from those in the oral cavity and colon. BMC Microbiol. 17, 160. doi:10.1186/s12866-017-1059-6 28716079 PMC5513040

[R176] Suzuki T, Sutani T, Nakai H, Shirahige K, and Kinoshita S (2020). The microbiome of the meibum and ocular surface in healthy subjects. Invest. Ophthalmol. Vis. Sci 61, 18. doi:10.1167/iovs.61.2.18 PMC7326502 32053729

[R177] Swidsinski A, Dörffel Y, Loening-Baucke V, Theissig F, Rückert JC, Ismail M, (2011). Acute appendicitis is characterised by local invasion with Fusobacterium nucleatum/necrophorum. Gut 60, 34–40. doi:10.1136/gut.2009.191320 19926616

[R178] Tanaka M, and Nakayama J (2017). Development of the gut microbiota in infancy and its impact on health in later life. Allergol. Int 66, 515–522. doi:10.1016/j.alit.2017.07.010 28826938

[R179] Teo SM, Mok D, Pham K, Kusel M, Serralha M, Troy N, (2015). The infant nasopharyngeal microbiome impacts severity of lower respiratory infection and risk of asthma development. Cell Host Microbe 17, 704–715. doi:10.1016/j.chom.2015.03.008 25865368 PMC4433433

[R180] Tian P, Zou R, Song L, Zhang X, Jiang B, Wang G, (2019). Ingestion of Bifidobacterium longum subspecies infantis strain CCFM687 regulated emotional behavior and the central BDNF pathway in chronic stress-induced depressive mice through reshaping the gut microbiota. Food Funct. 10, 7588–7598. doi:10.1039/c9fo01630a 31687714

[R181] Tito RY, Knights D, Metcalf J, Obregon-Tito AJ, Cleeland L, Najar F, (2012). Insights from characterizing extinct human gut microbiomes. PLoS One 7, e51146. doi:10.1371/journal.pone.0051146 23251439 PMC3521025

[R182] Tun HM, Konya T, Takaro TK, Brook JR, Chari R, Field CJ, (2017). Exposure to household furry pets influences the gut microbiota of infant at 3–4 months following various birth scenarios. Microbiome 5, 40. doi:10.1186/s40168-017-0254-x 28381231 PMC5382463

[R183] Turnbaugh PJ, Ley RE, Hamady M, Fraser-Liggett CM, Knight R, Gordon JI, (2007). The human microbiome project. Nature 449, 804–810. doi:10.1038/nature06244 17943116 PMC3709439

[R184] Valenti P, Rosa L, Capobianco D, Lepanto MS, Schiavi E, Cutone A, (2018). Role of Lactobacilli and lactoferrin in the mucosal cervicovaginal defense. Front. Immunol 9, 376. doi:10.3389/fimmu.2018.00376 29545798 PMC5837981

[R185] Valles-Colomer M, Falony G, Darzi Y, Tigchelaar EF, Wang J, Tito RY, (2019). The neuroactive potential of the human gut microbiota in quality of life and depression. Nat. Microbiol 4, 623–632. doi:10.1038/s41564-018-0337-x 30718848

[R186] Vargas-Robles D, Morales N, Rodríguez I, Nieves T, Godoy-Vitorino F, Alcaraz LD, (2020). Changes in the vaginal microbiota across a gradient of urbanization. Sci. Rep 10, 12487. doi:10.1038/s41598-020-69111-x 32719372 PMC7385657

[R187] Vera-Urbina F, Dos Santos-Torres MF, Godoy-Vitorino F, and Torres-Hernández BA (2022). The gut microbiome may help address mental health disparities in Hispanics: A narrative review. Microorganisms 10, 763. doi:10.3390/microorganisms10040763 35456813 PMC9029366

[R188] Wallace BD, Wang H, Lane KT, Scott JE, Orans J, Koo JS, (2010). Alleviating cancer drug toxicity by inhibiting a bacterial enzyme. Science 330, 831–835. doi:10.1126/science.1191175 21051639 PMC3110694

[R189] Wen Z, Xie G, Zhou Q, Qiu C, Li J, Hu Q, (2018). Distinct nasopharyngeal and oropharyngeal microbiota of children with influenza A virus compared with healthy children. Biomed. Res. Int 2018, 6362716. doi:10.1155/2018/6362716 PMC6276510 30581863

[R190] Wibowo MC, Yang Z, Borry M, Hübner A, Huang KD, Tierney BT, (2021). Reconstruction of ancient microbial genomes from the human gut. Nature 594, 234–239. doi:10.1038/s41586-021-03532-0 33981035 PMC8189908

[R191] Winglee K, Howard AG, Sha W, Gharaibeh RZ, Liu J, Jin D, (2017). Recent urbanization in China is correlated with a Westernized microbiome encoding increased virulence and antibiotic resistance genes. Microbiome 5, 121. doi:10.1186/s40168-017-0338-7 28915922 PMC5603068

[R192] Wu GD, Chen J, Hoffmann C, Bittinger K, Chen Y-Y, Keilbaugh SA, (2011). Linking long-term dietary patterns with gut microbial enterotypes. Science 334, 105–108. doi:10.1126/science.1208344 21885731 PMC3368382

[R193] Xie F-J, Zhang Y-P, Zheng Q-Q, Jin H-C, Wang F-L, Chen M, (2013). *Helicobacter pylori* infection and esophageal cancer risk: An updated meta-analysis. World J. Gastroenterol 19, 6098–6107. doi:10.3748/wjg.v19.i36.6098 24106412 PMC3785633

[R194] Ye L, and Liddle RA (2017). Gastrointestinal hormones and the gut connectome. Curr. Opin. Endocrinol. Diabetes Obes 24, 9–14. doi:10.1097/MED.0000000000000299 27820704 PMC5815400

[R195] Yin G, Li JF, Sun YF, Ding X, Zeng JQ, Zhang T, (2019). [Fecal microbiota transplantation as a novel therapy for severe psoriasis]. Zhonghua Nei Ke Za Zhi 58, 782–785. doi:10.3760/cma.j.issn.0578-1426.2019.10.011 31594178

[R196] Zakerihamidi M, Latifnejad Roudsari R, and Merghati Khoei E (2015). Vaginal delivery vs. Cesarean section: A focused ethnographic study of women’s perceptions in the North of Iran. Int. J. Community Based Nurs. Midwifery 3, 39–50. 25553333 PMC4280556

[R197] Zhang Z, and Li D (2018). Thermal processing of food reduces gut microbiota diversity of the host and triggers adaptation of the microbiota: Evidence from two vertebrates. Microbiome 6, 99. doi:10.1186/s40168-018-0471-y 29855351 PMC5984331

[R198] Zhou X, Brown CJ, Abdo Z, Davis CC, Hansmann MA, Joyce P, (2007). Differences in the composition of vaginal microbial communities found in healthy Caucasian and black women. ISME J. 1, 121–133. doi:10.1038/ismej.2007.12 18043622

[R199] Zilber-Rosenberg I, and Rosenberg E (2008). Role of microorganisms in the evolution of animals and plants: The hologenome theory of evolution. FEMS Microbiol. Rev 32, 723–735. doi:10.1111/j.1574-6976.2008.00123.x 18549407

[R200] Zimmermann M, Zimmermann-Kogadeeva M, Wegmann R, and Goodman AL (2019). Mapping human microbiome drug metabolism by gut bacteria and their genes. Nature 570, 462–467. doi:10.1038/s41586-019-1291-3 31158845 PMC6597290

[R201] Zoetendal EG, Raes J, van den Bogert B, Arumugam M, Booijink CCGM, Troost FJ, (2012). The human small intestinal microbiota is driven by rapid uptake and conversion of simple carbohydrates. ISME J. 6, 1415–1426. doi:10.1038/ismej.2011.212 22258098 PMC3379644

[R202] Zwittink RD, Renes IB, van Lingen RA, van Zoeren-Grobben D, Konstanti P, Norbruis OF, (2018). Association between duration of intravenous antibiotic administration and early-life microbiota development in late-preterm infants. Eur. J. Clin. Microbiol. Infect. Dis 37, 475–483. doi:10.1007/s10096-018-3193-y 29368074 PMC5816780

